# Supplementary material for: Photonic Materials Cloud: An Online Interactive Open Tool for Creating, Comparing, and Testing Photonic Materials
Source: Nanomaterials (Basel). 2022 Jul 28;12(15):2585. doi: 10.3390/nano12152585 (PMC9370397; doi:10.3390/nano12152585)
Supplement: Supplementary file 1 [file nanomaterials-12-02585-s001.zip › nanomaterials-1825644-supplementary.pdf]

Supplementary Information: Photonic Materials Cloud: An Online Interactive Open Tool for Creating, Comparing, and Testing Photonic Materials

M. T. Korsa[1,†], Petersen[1,†], Neda Rahmani[3], Alireza Shabani[3], Yogendra Mishra[2], and Jost Adam[1]

[1] Computational Materials Group, SDU Centre for Photonics Engineering, Mads Clausen Institute, University of Southern Denmark, DK-5320 Odense, Denmark
[2] Smart Materials, NanoSyd, Mads Clausen Institute, University of Southern Denmark, DK-6400 Sønderborg, Denmark
[3] Mechatronics, Department of Mechanical and Electrical Engineering, University of Southern Denmark, DK-6400 Sønderborg, Denmark
[†] These authors contributed equally to the work.

This supplementary material constitutes a step-by-step tutorial introduction to the Photonic Materials Cloud [2].

Contents

1 Introduction **2**

2 Select Materials Tab **2**
2.1 Basic Workflow . . . 2
2.2 Exporting and Importing a List of Materials . . . 2
2.3 Material Model: Upload a File . . . 5
2.4 Material Model: Select File from Library . . . 5
2.5 Material Model: Drude-Lorentz Parameters . . . 6
2.6 Material Model: Constant Refractive Index . . . 7

3 Compare Materials Tab **10**
3.1 Basic Workflow . . . 10

4 Particle Scattering Tab **11**
4.1 Basic Workflow . . . 11

5 Thin Film Tab **12**
5.1 Basic Workflow . . . 12
5.2 Add Single Layer Option . . . 13
5.3 Add Recurring Layer Option . . . 13
5.4 Import Layers Option . . . 14

6 Examples **16**
6.1 Methods of specifying materials and comparison, AZO . . . 16
6.1.1 Assign materials . . . 16
6.1.2 Compare Materials . . . 16
6.2 Mie scattering results, ZrN . . . 20
6.2.1 Assign materials . . . 20
6.2.2 Create Dispersed Particle System . . . 23
6.2.3 Compute Mie-Scattering Results . . . 23
6.3 Thin Film Results, ZrN . . . 24
6.3.1 Assign Materials . . . 24
6.3.2 Create Thin Film Layer Sequence . . . 25
6.3.3 Compute Optical Properties . . . 25
6.4 The Bragg Reflector . . . 30
6.4.1 Assign materials . . . 30
6.4.2 Create Bragg reflector layer sequence . . . 30
6.4.3 Compute optical properties . . . 34

1 Introduction

This tutorial explains the workflow and gives examples of how to use the photonics materials cloud [2], which is an online calculation platform about photonic materials developed by the SDU Computational materials group [1]. The tool can be found on the groups' website and is for scientific and educational purposes. Using this tool, users can interactively create, browse, and compare photonic materials, dispersion, and light-matter interaction online.
The dashboard's functionality is currently threefold; the user can compare the refractive indices of two or more materials, calculate Mie scattering of shell-core particles dispersed in water, and - thin-film response of layered structures. These functionalities exist on each of their individual tabs.

2 Select Materials Tab

The first tab is the Select Materials Tab, which is the first tab the user should open since this tab is the entry for all subsequent calculations. This tab is used to create a list of up to 20 different materials via either database entries, file uploads, Drude-Lorentz parameters, or constant refractive indices. Each material's (n,k) dispersion curves can be previewed before assigning it to the list. The material list persists throughout the whole page and serves as the basis for the comparison and calculation tabs. Come back to this tab any time to update your basic list of materials.

2.1 Basic Workflow

The basic workflow of the select materials tab is described below - please refer to figure 1.

1. use the navigation tabs to easily switch to other tabs by clicking them. Navigate to the select materials tab by clicking 'Select Materials'
2. click any of the four card headers to unfold the one corresponding to the desired method of specifying a material model
3. depending on the material model card selected, the user will be presented with a card requiring the user to upload a file, select a library material from a drop-down menu or input material parameters
4. click 'preview material' when the unfolded card parameters are entered to load the material into the memory of the dashboard and preview the material
5. the materials' optical properties is plotted. Use the graph axis options to toggle between n/k and epsilon y-axis labels, wavelength and energy x-axis labels and the x-axis range to confirm the material model is as desired
6. click the drop-down to select, from 1 to 20, as which material number the previewed material should be stored Selecting and assigning a material number that is already occupied will override the previously assigned material
7. click 'Assign to material' to store the material in the list of materials, as the selected material number
8. the previewed material will appear in the list of materials at the selected number and can now be used on the other tabs to calculate optical responses and compare materials.

2.2 Exporting and Importing a List of Materials

Once a list of materials has been generated, often the user might want to reuse the same list of materials in future calculations. This can be accomplished using the export/import materials list on the select materials tab, described below - please refer to figure 2.

1. once the user has generated a list of materials, click the export materials list button.
2. the list of materials is downloaded as a .csv file which the user can save and distribute to collaborators.
3. the next time a user wants to use the dashboard with the previously generated list of materials, click the 'Drag and Drop or Select Files' button. Use the popup window to navigate to the previously exported .csv file and select it
4. once the file is selected, click import materials list and the materials in the .cvs file will appear in the list of materials - these materials can now be used for calculations on the other tabs

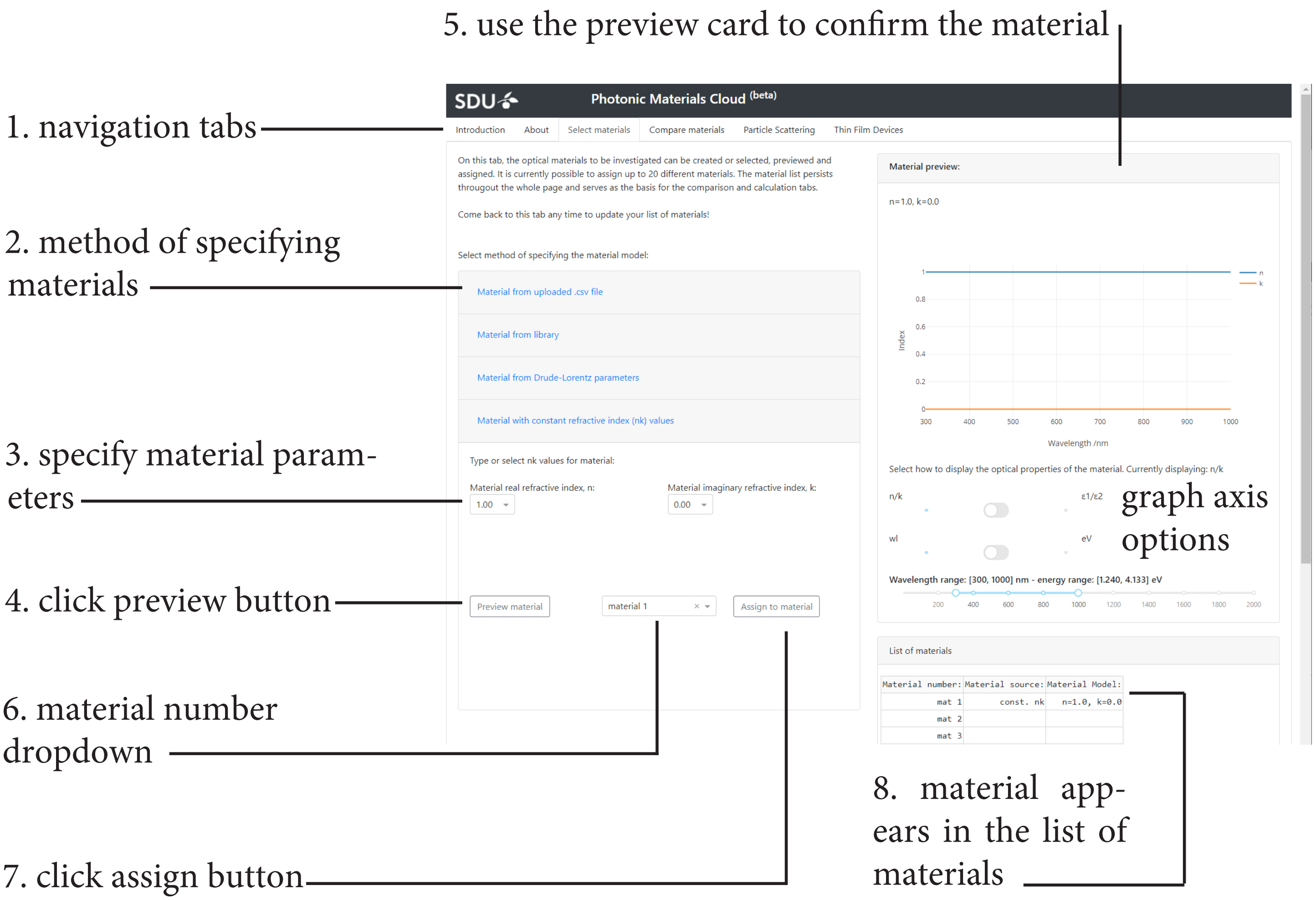

Figure 1: Select materials tab and -basic workflow

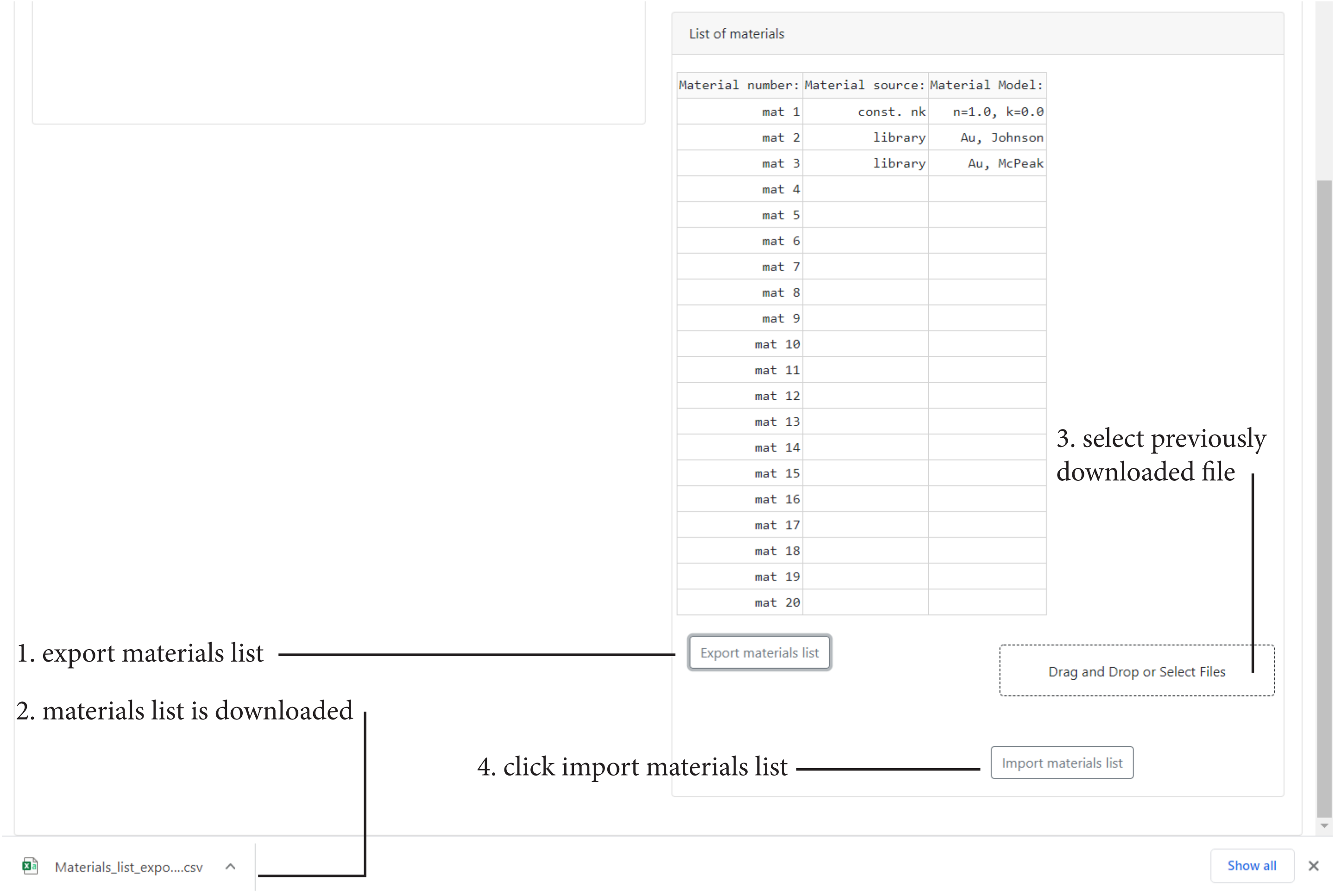

Figure 2: Select materials tab - exporting and importing list of materials

2.3 Material Model: Upload a File

Below is described the upload .csv file method of specifying a material model - please refer to figure 3.

1. click the 'Material from uploaded file' header to unfold the card
2. click the 'Drag and Drop or Select Files' button
3. Use the popup window to navigate to the material model .csv file and select it. NOTE: it is important that the first row of the file is the column names, and they should be named 'wavelength', 'n' and 'k'
4. select the units of the wavelengths entries of the uploaded file. Units of manometers [nm] by default
5. click 'preview material' to load the material into the memory of the dashboard and preview the material
6. click the drop-down to select, from 1 to 20, which material number the previewed material should be stored.
7. click 'Assign to material' to store the material in the list of materials, as the selected material number - this materials can now be used for calculations on the other tabs

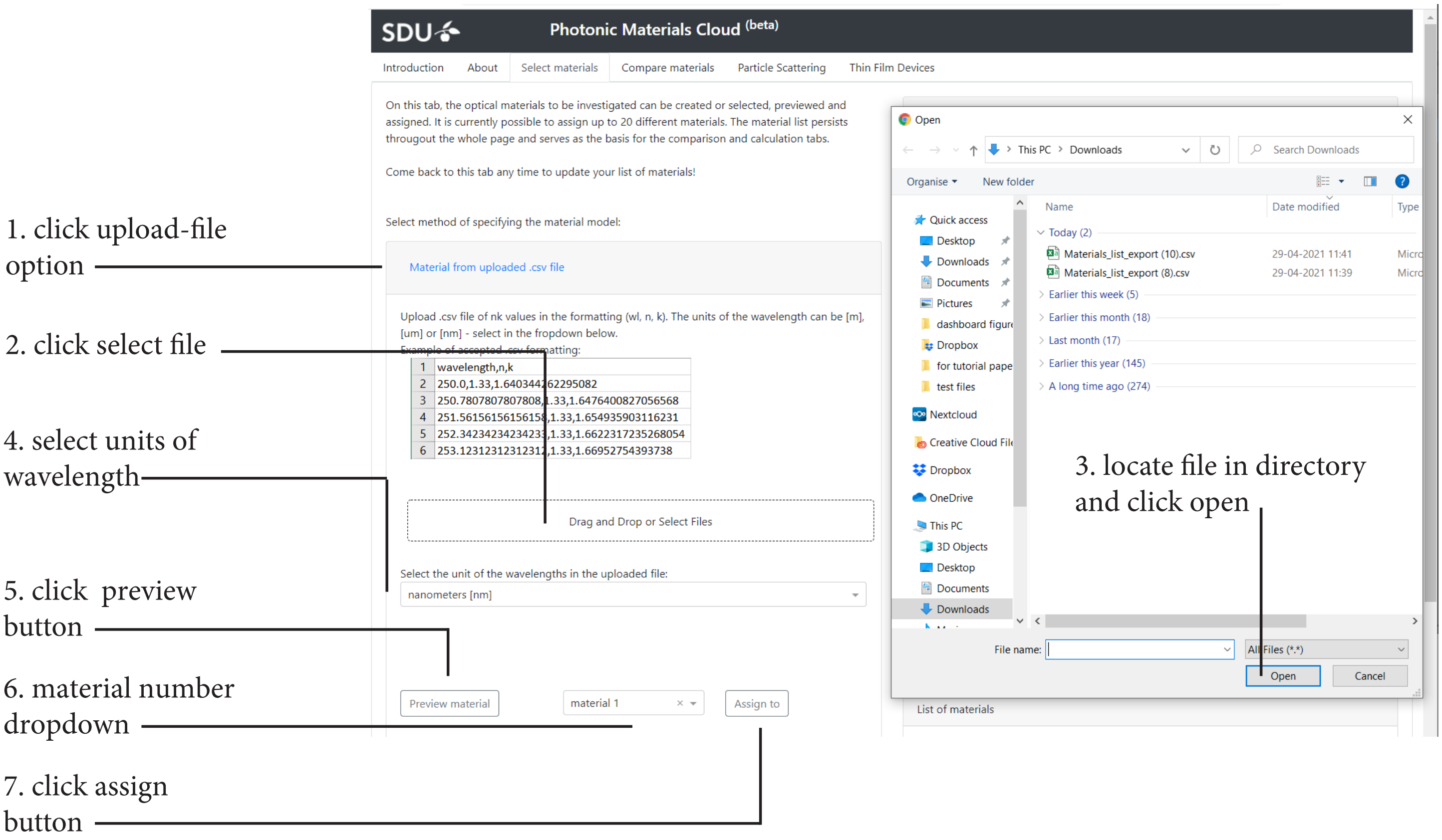

Figure 3: Select materials workflow, upload file option

2.4 Material Model: Select File from Library

Below is described the select file from library method of specifying a material model - please refer to figure 4.

1. click the 'Material from library' header to unfold the card
2. use the drop-downs to navigate and select a material from the library. The drop-downs must be selected in order from top to bottom, where the first drop-down is used to specify the class of materials, the next is the materials itself, and the final drop-down selects the material model data-set.
3. click 'preview material' to load the material into the memory of the dashboard and preview the material
4. click the drop-down to select, from 1 to 20, which material number the previewed material should be stored.
5. click 'Assign to material' to store the material in the list of materials, as the selected material number - this materials can now be used for calculations on the other tabs

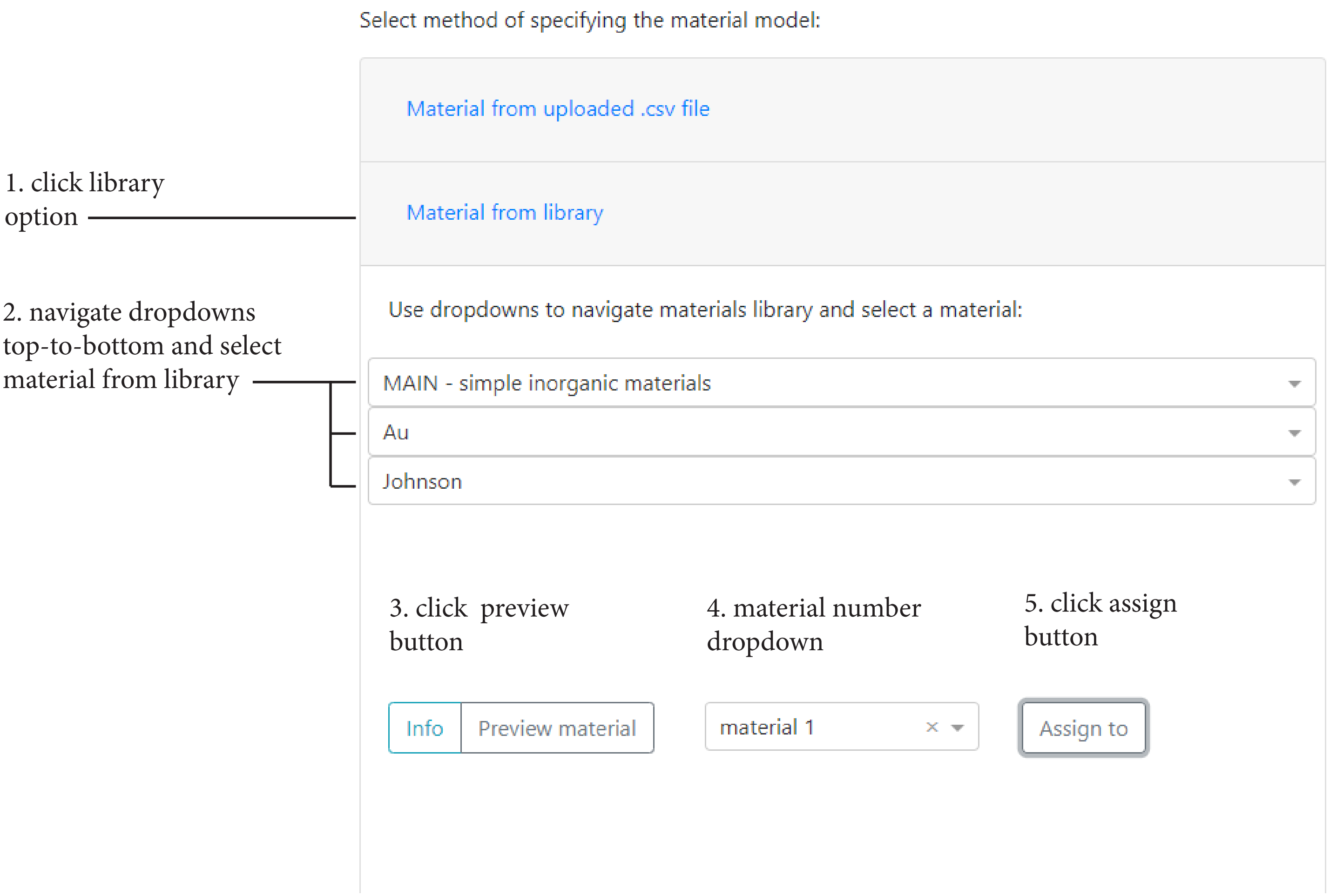

Figure 4: Select materials workflow, library option

2.5 Material Model: Drude-Lorentz Parameters

Below is described the Drude-Lorentz parameters method of specifying a material model - please refer to figure 5.

1. click the 'Material from Drude-Lorentz Parameters' header to unfold the card
2. enter the Drude plasma frequency and -damping factor of the material into the boxes in the first sub-card
3. use the toggle button in the second sub-card to select if the magnitude of the Lorentzian peaks should be specified in terms of Lorentzian peak coefficients or -strengths. The respective formulas used to estimate the refractive index can be seen on the right.
4. enter at least one (the first) set of Lorentzian parameters. Be careful to differentiate between Lorentzian peak coefficients or -strengths in the third box, depending on which is selected in (2)
5. (optional) specify further sets of Lorentzian parameters, up to a total of 10, to incorporate more features of the refractive index model. This is done by:
   5.1. click the 'Set (2-10) of parameters (optional)' header to unfold the sub-card
   5.2. enter at the (2nd-10th) set of Lorentzian parameters. Again, be careful to differentiate between Lorentzian peak coefficients or -strengths in the third box, depending on which is selected in (2)
   5.3. repeat steps (5) as many times as desired, up to a maximum of 10 sets of Lorentzian parameters
6. click 'preview material' to load the material into the memory of the dashboard and preview the material
7. click the drop-down to select, from 1 to 20, as which material number the previewed material should be stored.
8. click 'Assign to material' to store the material in the list of materials, as the selected material number - this materials can now be used for calculations on the other tabs

2.6 Material Model: Constant Refractive Index

Below is described the constant refractive index method of specifying a material model - please refer to figure 6.

1. click the 'Material with constant refractive index (nk) values' header to unfold the card
2. use the boxes to type (or pick) values for the real- and imaginary part of the refractive index (n- and k value). The values must be between 0 and 5
3. click 'preview material' to load the material into the memory of the dashboard and preview the material
4. click the drop-down to select, from 1 to 20, which material number the previewed material should be stored.
5. click 'Assign to material' to store the material in the list of materials, as the selected material number - this materials can now be used for calculations on the other tabs

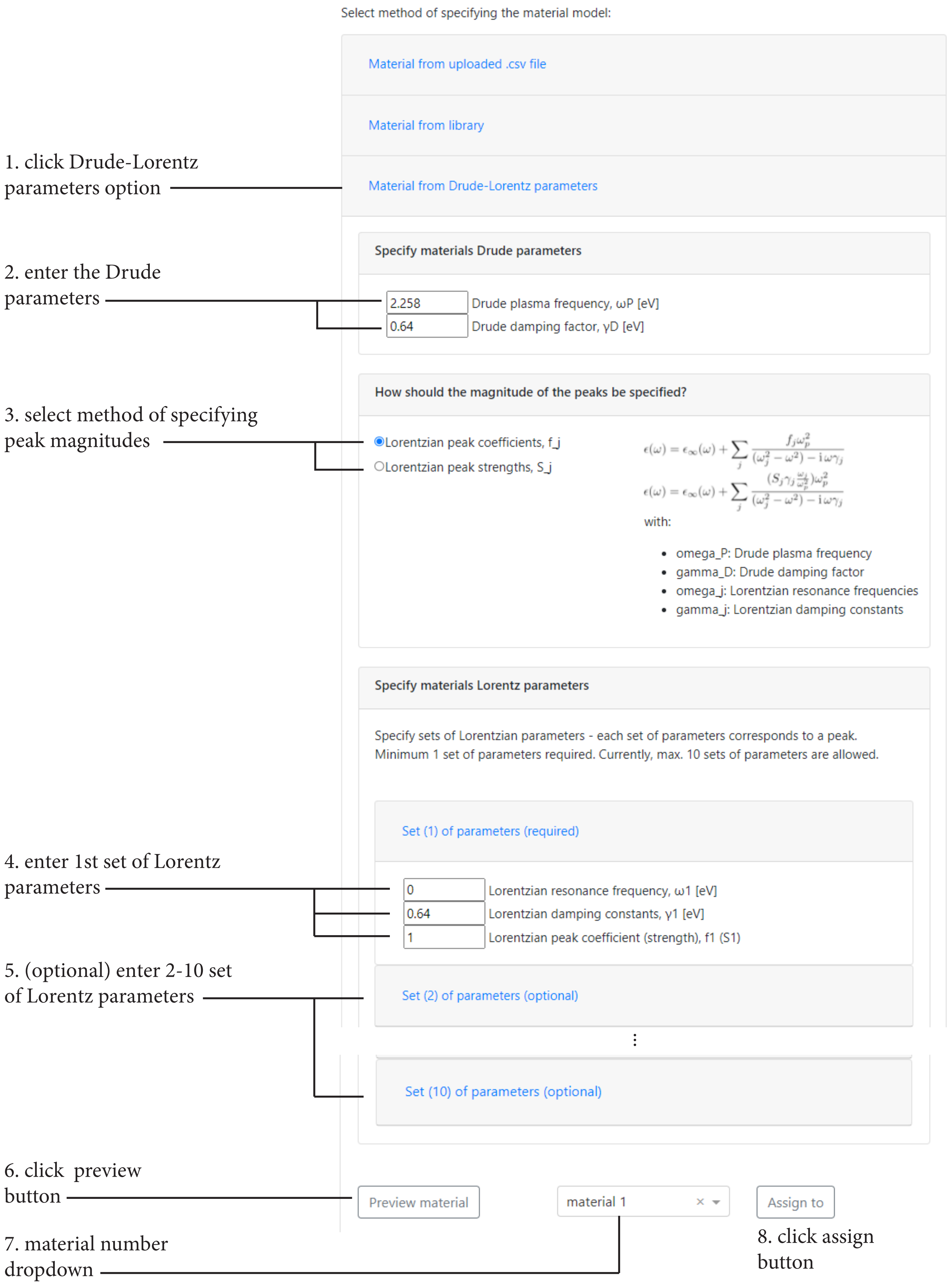

Figure 5: Select materials workflow, Drude-Lorentz option

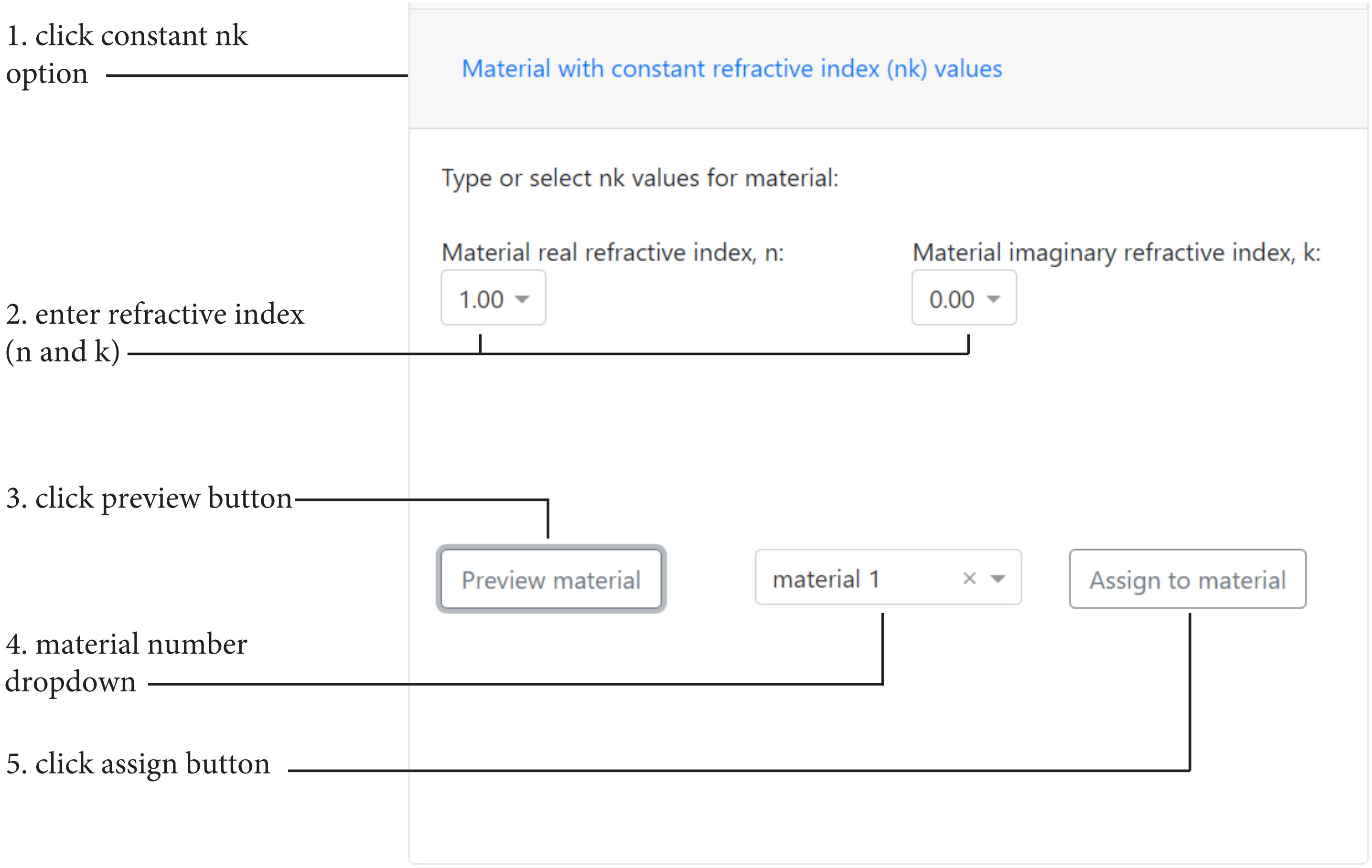

Figure 6: Select materials workflow, constant refractive index option

3 Compare Materials Tab

The compare materials tab can be used to compare the materials which have been assigned on the select materials tab. Select up to 20 materials to be plotted and use the interactive graph to investigate obvious and subtle differences in the optical characteristics of the materials. The refractive indices of the plotted materials compared can be downloaded to the users computers in .csv format.
Remember to assign materials or import materials on the select materials tab, making the materials eligible for selection on this tab.

3.1 Basic Workflow

The basic workflow of the compare materials tab is described below - please refer to figure 7.

1. use the navigation tabs to easily switch to other tabs by clicking them. Navigate to the compare materials tab by clicking 'Compare Materials'
2. click the 'select materials to compare' drop-down, revealing all the materials created (or imported) on the select materials tab previously. Click the materials which should be compared in the drop-down. It is possible to compare many materials, up to 20 different ones.
3. the materials selected in the compare materials drop-down is plotted in the interactive graph
4. the wavelength range and discretization of the refractive index plot can be adjusted using the plotting parameters
5. the unit of the x- and y-axis can be selected using the toggle buttons
6. click the 'List of materials' header to expand a card containing the full list of materials
7. click the 'save refractive index data' button to download a .csv file containing all the selected materials which are plotted.

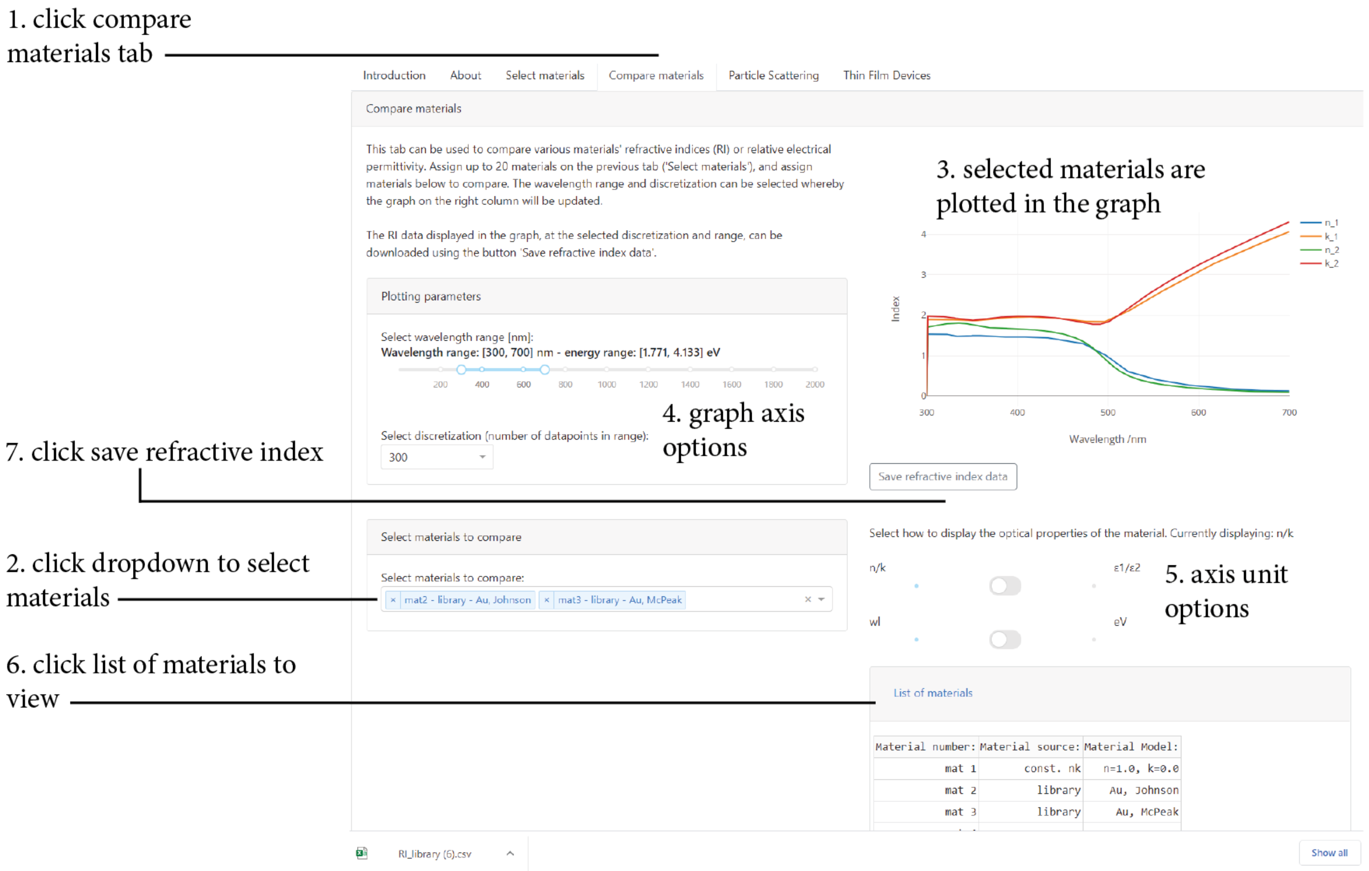

Figure 7: Compare materials basic workflow.

4 Particle Scattering Tab

The particle scattering tab can be used to compute the Mie scattering of spherical, coated (or non-coated) nano-particles dispersed in a medium.
The core, shell, and surrounding medium materials can be freely picked from the material list. The dashboard calculates (forward) scattering, absorption, and extinction dispersion interactively, with variable wavelength/energy range and sampling rate. The results can be displayed over energy or wavelength and in terms of refractive indices or electric permittivity.

4.1 Basic Workflow

The basic workflow of the particle scattering tab is described below - please refer to figure 8.

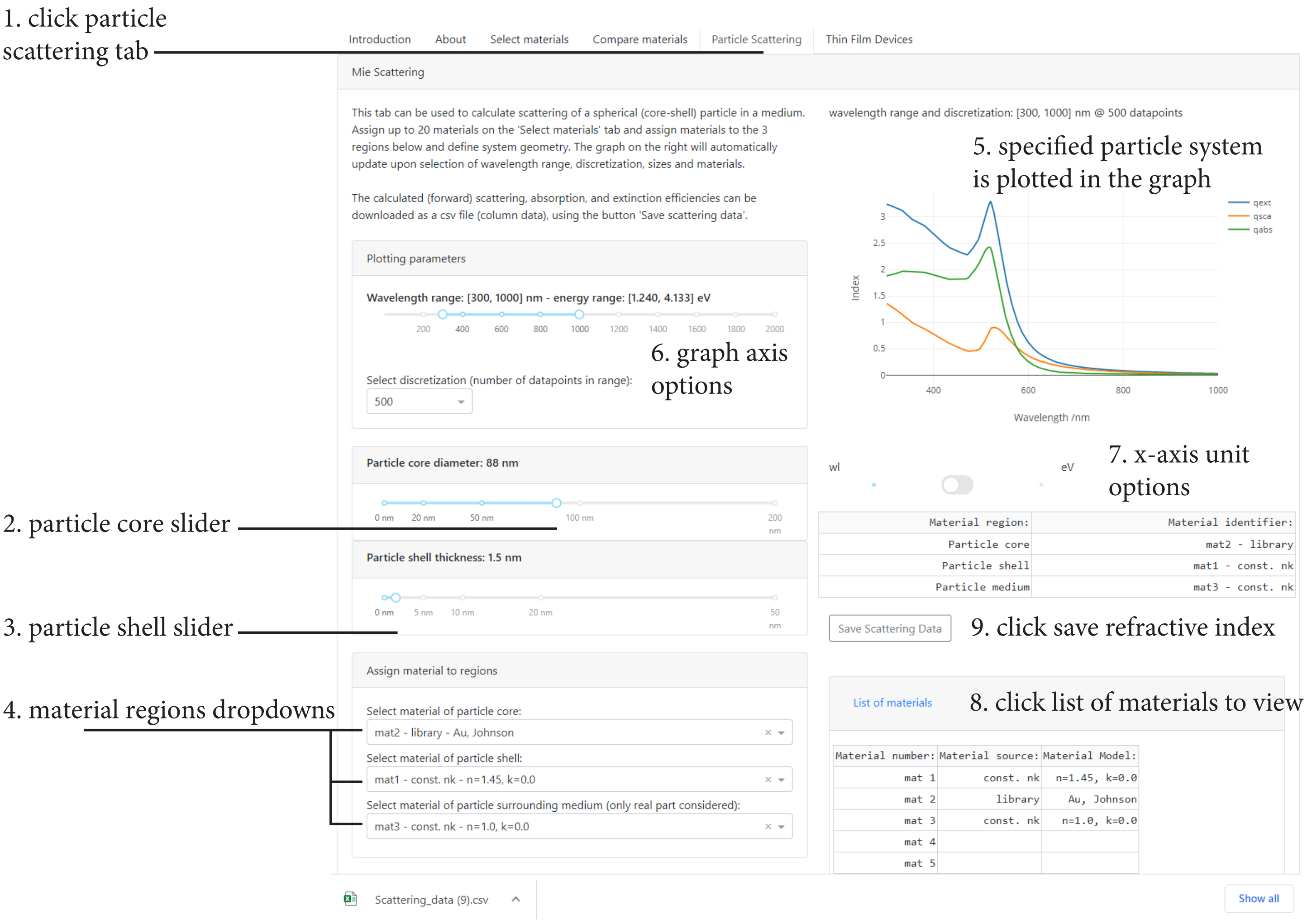

Figure 8: Particle scattering basic workflow.

1. use the navigation tabs to easily switch to other tabs by clicking them. Navigate to the particle scattering tab by clicking 'Particle Scattering'
2. use the particle core diameter slider to specify the diameter of the spherical nanoparticle in units of nanometers
3. use the shell thickness slider to specify the thickness of the nanoparticle coating of the spherical nanoparticle in units of nanometers
4. assign materials to the core-, shell- and surrounding medium regions
   4.1. click the 'select material of particle core' drop-down, revealing all the materials created (or imported) on the select materials tab previously. Click the material which should be assigned to the particle core in the drop-down.

4.2. click the 'select material of particle shell' drop-down, revealing all the materials created (or imported) on the select materials tab previously. Click the material which should be assigned to the particle shell in the drop-down.

4.3. click the 'select material of particle surrounding medium' drop-down, revealing all the materials created (or imported) on the select materials tab previously. Click the material which should be assigned to the particle surrounding medium in the drop-down. Note that only the real part of the refractive index is considered for this material region

5. the dashboard calculates the Mie scattering of the system with the given parameters and materials and plots the scattering, absorption and extinction in the interactive graph

6. the wavelength range and discretization of the scattering plot can be adjusted using the plotting parameters

7. the unit of the x-axis can be selected using the toggle buttons

8. click the 'List of materials' header to expand a card containing the full list of materials

9. click the 'save scattering data' button to download a .csv file containing the scattering data of the configuration which is plotted.

5 Thin Film Tab

The thin film tab admits the user to calculate the reflection, transmission, and absorption profiles of multi-layer thin-film stacks: The basis for the design of photonic thin-film devices. This calculation is based on the thin film structure, which the user generates on this tab and using the materials which is defined on the previous tab.

5.1 Basic Workflow

The basic workflow of the tab is described below - please refer to figure 9.

1. use the navigation tabs to easily switch to other tabs by clicking them. Navigate to the thin film tab by clicking 'Thin Film Devices'

2. click one of the 3 headers, 'add single layer', 'add recurring layer' or 'import layers', to reveal their respective cards. Here, the user can add the materials- and thicknesses of layers either one at a time, a recurring number of times, a combination of the two or by importing a previously exported sequence of layers. Each option is described further in the sections to follow

3. each time a layers is added, a graphic updates which displays the sequence of the defined layers, their relative thicknesses, and an arrow indicating the incidence light vector. The colour of the layers correspond to each layers real part of its refractive index (average if not constant n)

4. click the 'List of Layers' header to expand and hide a card revealing the sequence of layers. From here, the current list of layers can be cleared by clicking the 'clear layers' button

5. click the 'Export Layers' button in the expanded card to download a .csv file containing the current sequence of layers. This file can be saved and shared with collaborators, who can use the 'import layers' to start off where one left previously without having to redefine all the materials and layers again

6. click one of the three headers, 'Wavelength sweep, fixed incidence angle', 'Wavelength fixed, sweep incidence angle' or 'Wavelength sweep, sweep incidence angle', depending on the type of results the user is looking for, revealing their respective cards. Here, the user can specify the incident angle, -wavelength and/or their ranges of the light as well as the discretization and light polarization

7. click the 'Calculate' button to calculate the reflection, transmission, and absorption profiles of the generated thin film structure

8. the optical profile of the structure is plotted in interactive graphs

9. click the 'Save 1D Optical Data'- or 'Save 1D Optical Data' buttons, depending on the type of results card selected, to save the data as a .csv file.

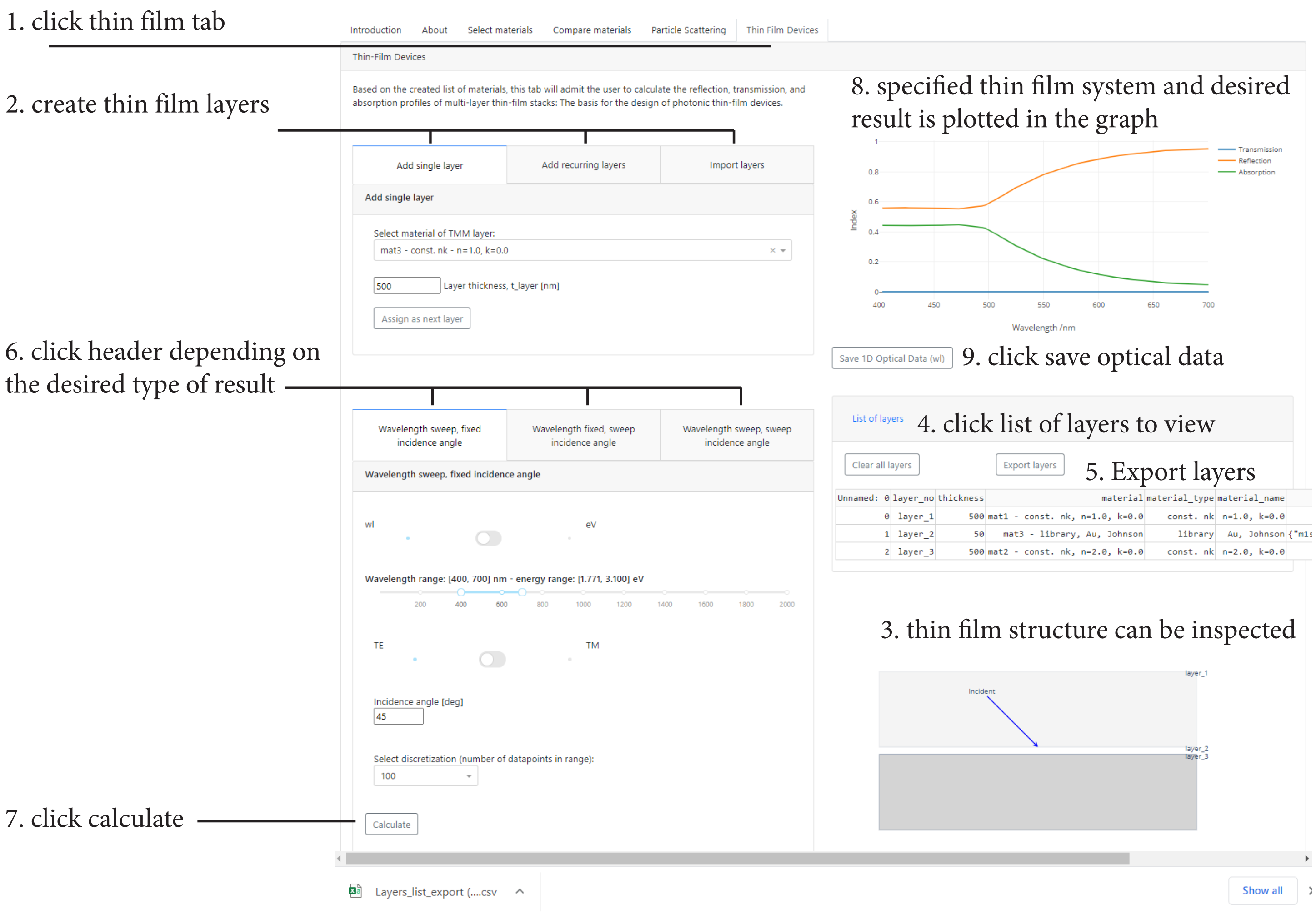

Figure 9: Thin film basic workflow.

5.2 Add Single Layer Option

This section describes the method of assigning a single layer at a time to the thin film structure - please refer to figure 10.

1. click card header 'add single layer' to reveal the card
2. click the 'select material of TMM layer' drop-down, revealing all the materials created (or imported) on the select materials tab previously. Click the material which should be assigned to the first TMM layer in the drop-down.
3. enter the thickness of the current TMM layer in units of nanometers
4. click the 'assign as next layer' button to confirm and load the specified layer as the next layer
5. repeat steps 2-4 to assign as many layers as desired

5.3 Add Recurring Layer Option

The option to add recurring layers is not currently implemented. In the meantime, we recommend that the user:

1. adds the unique layers of the desired recurring configuration using the 'add single layers' option
2. exports the generated sequence of layers (as .csv)
3. opens the .csv file, copies the sequence of recurring layers the desired number of times and saves the file
4. uses the 'import layers' option to import the new .csv file with the recurring layers

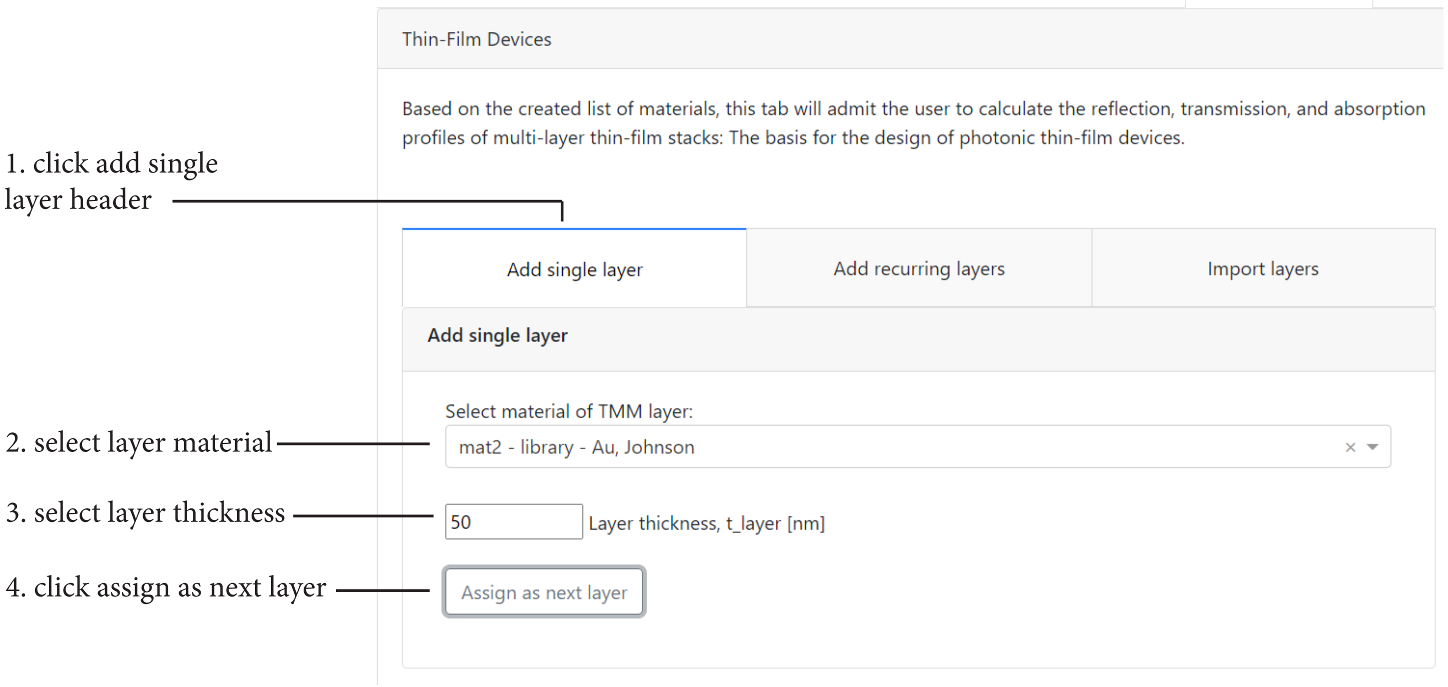

Figure 10: Thin film workflow, add single layer.

5.4 Import Layers Option

This section describes the method of importing a layer sequence to the thin film structure - please refer to figure 11.

1. click card header 'import layers' to reveal the card
2. click the 'Drag and Drop or Select Files' to open the dialogue window
3. navigate to the previously exported .csv file containing the sequence of layers and select it by clicking it
4. a preview of the sequence of layers is displayed
5. click the 'import layers' button to load the sequence of layers into the dashboard

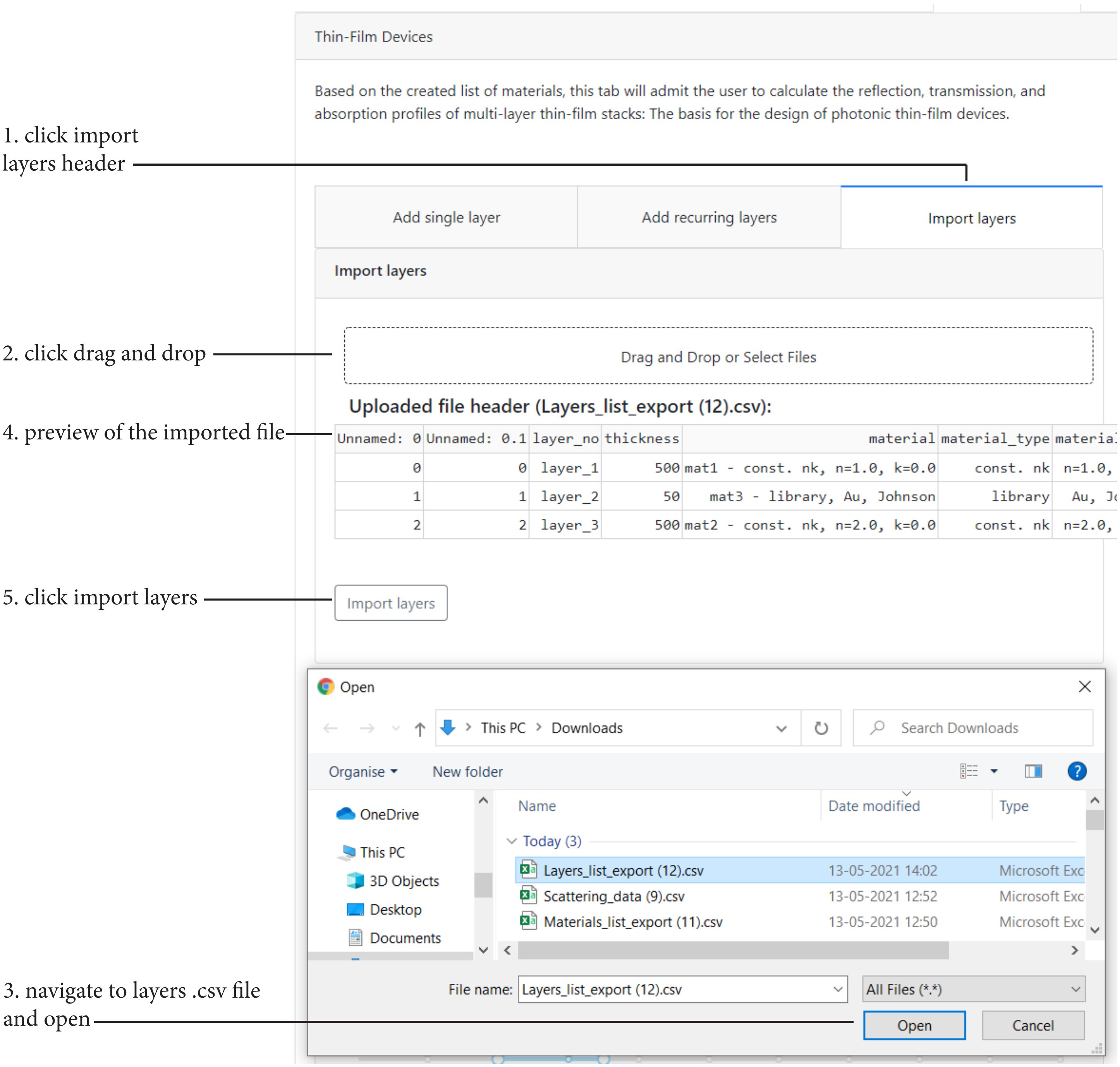

Figure 11: Thin film workflow, upload layers.

6 Examples

In the following, some examples are provided that the user can recreate using the dashboard.

6.1 Methods of specifying materials and comparison, AZO

This example demonstrates the Drude-Lorentz- and the upload file methods of specifying materials on the select materials tab - in this example, AZO6 is used as the test material. Also, the compare materials tab is used to compare the various ways of specifying the material models.

6.1.1 Assign materials

First, we need to assign the materials using the 'select materials' tab on the dashboard. This example uses the AZO6 material. First, we will specify the material using Drude-Lorentz parameters. Next, we will compare it to a file containing nk values - the user can follow this example if such an nk file is accessible. Assign the material in the following way.

1. navigate to the select materials tab by clicking 'Select Materials'
2. create and store AZO6 material as material 1 (figure 12):
   - 2.1. click the 'Material from Drude-Lorentz parameters' header to unfold the card
   - 2.2. specify the Drude parameters for AZO6 - enter $\omega_P = 2.193\,\mathrm{eV}$ as the Drude plasma frequency, $\epsilon_\infty = 3.5\,\mathrm{F/m}$ as the electric permittivity at the high-frequency limit and $\gamma_D = 0.05\,\mathrm{eV}$ as the Drude damping factor
   - 2.3. choose to specify the Lorentzian peak magnitudes as Lorentzian peak coefficients, f_j
   - 2.4. specify set 1 of Lorentz parameters according to table 1:
     - 2.4.1. fill set (1) of parameters with entries from row no. 2 in table 1:
     - 2.4.2. enter $\omega_1 = 0.294\,\mathrm{eV}$ as the first Lorentzian resonance frequency
     - 2.4.3. enter $\gamma_1 = 0.213\,\mathrm{eV}$ as the first Lorentzian damping constant
     - 2.4.4. enter $f_1 = 0.243$ as the first Lorentzian peak coefficient
   - 2.5. repeat steps 3.4 to fill sets 2-8 of Lorentzian parameters using rows 3-9 in table 2
   - 2.6. click 'preview material'
   - 2.7. click the drop-down to select, from 1 to 20, which material number the previewed material should be stored. Select 'material 1'
   - 2.8. click 'Assign to material'
3. upload comparison nk file containing AZO6 refractive index (figure 13):
   - 3.1. click the 'Material from uploaded file' header to unfold the card
   - 3.2. click the 'Drag and Drop or Select Files' button
   - 3.3. Use the popup window to navigate to the material model .csv file and select it
   - 3.4. select the units of the wavelengths entries of the uploaded file
   - 3.5. click 'preview material' to load the material into the memory of the dashboard and preview the material
   - 3.6. click the drop-down to select, from 1 to 20, which material number the previewed material should be stored. Select 'material 2'
   - 3.7. click 'Assign to material'

6.1.2 Compare Materials

Next, we can compare the material models - the workflow is described below (figure 14).

1. navigate to the compare materials tab by clicking 'Compare Materials'
2. select the wavelength range $\gamma = 200\text{-}1600\,\mathrm{nm}$
3. select 500 for the democratization
4. click the 'select materials to compare' drop-down and select 'mat 2 - DL params...' and 'mat2 - file upload'
5. toggle ϵ_1/ϵ_2

Table 1: The optical parameters of Drude-Lorentz model for AZO6 obtained from DFT-GGA calculations [3]

Resonance j	ω_j [eV]	γ_j [eV]	f_j
1	0	0.05	1
2	0.294	0.213	0.243
3	4.810	0.286	0.186
4	5.258	0.433	0.351
5	5.863	0.613	0.597
6	6.607	0.741	0.890
7	7.449	0.816	1.237
8	8.159	0.772	1.281
9	8.790	0.791	2.728

6. toggle eV
7. inspect results

As we can see in figure 14, the two methods of specifying the AZO material have very similar optical properties to the experimental data nk file, confirming the Drude-Lorentz parameters method.

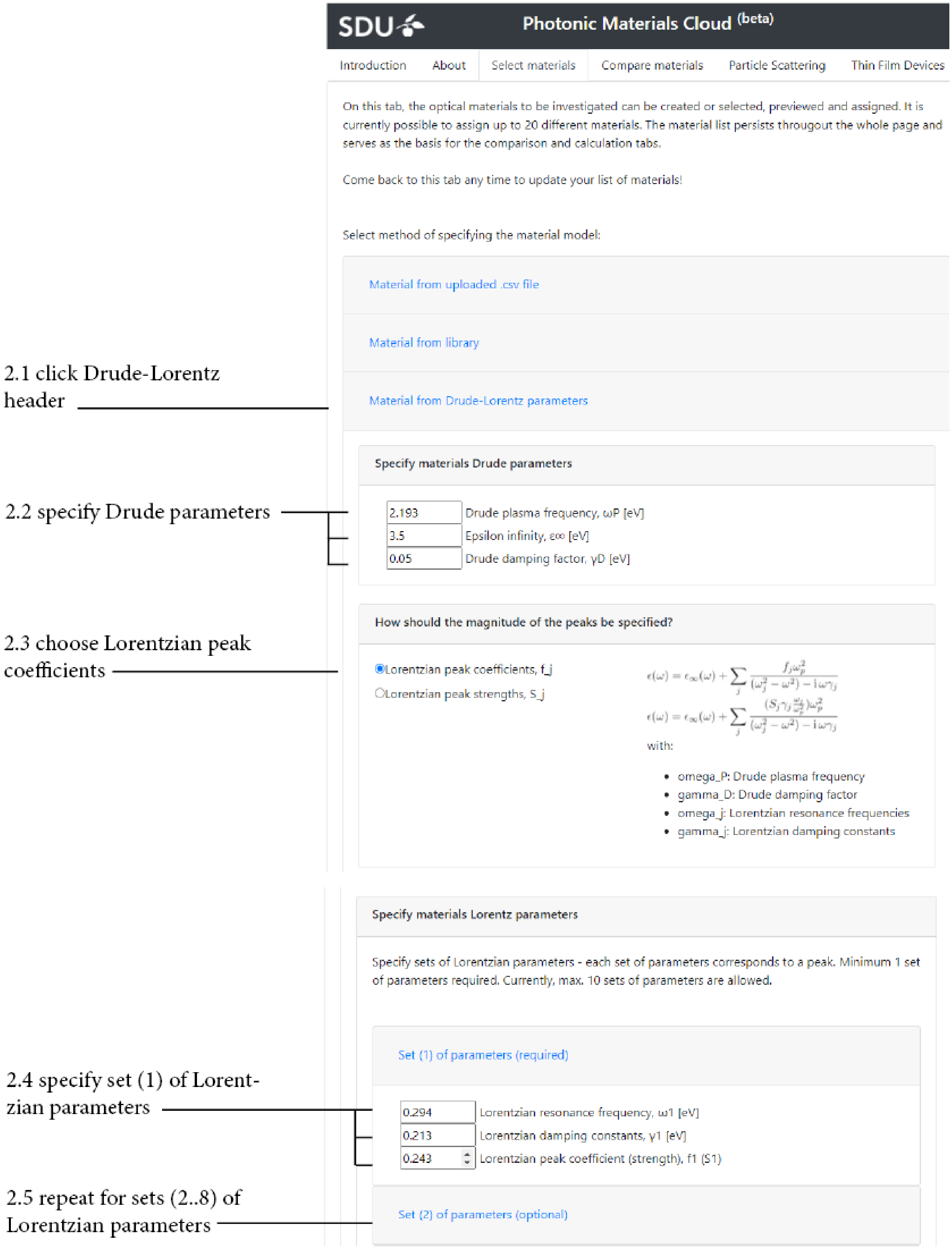

Figure 12: Assigning AZO6 Drude parameters.

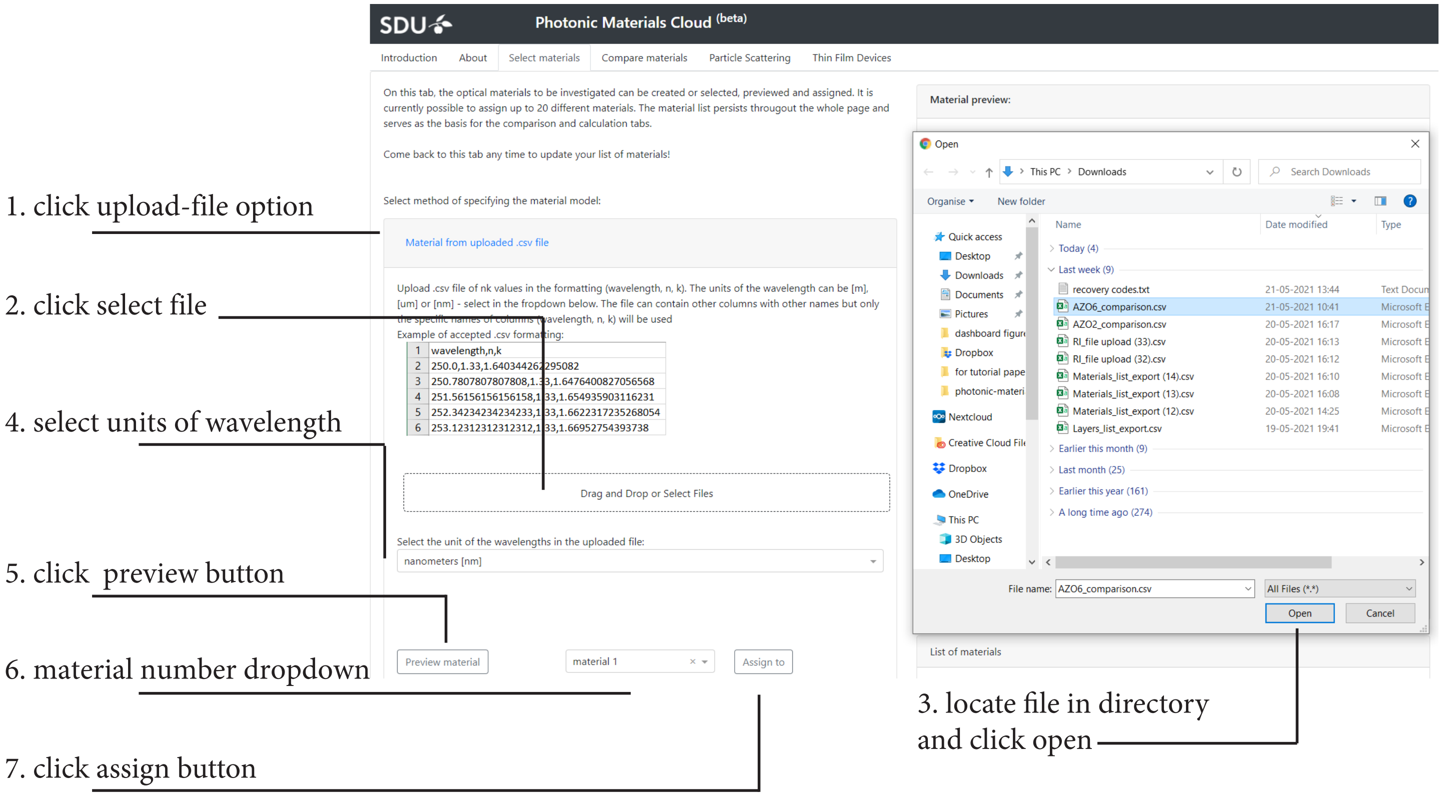

Figure 13: Uploading .csv AZO6 material parameters.

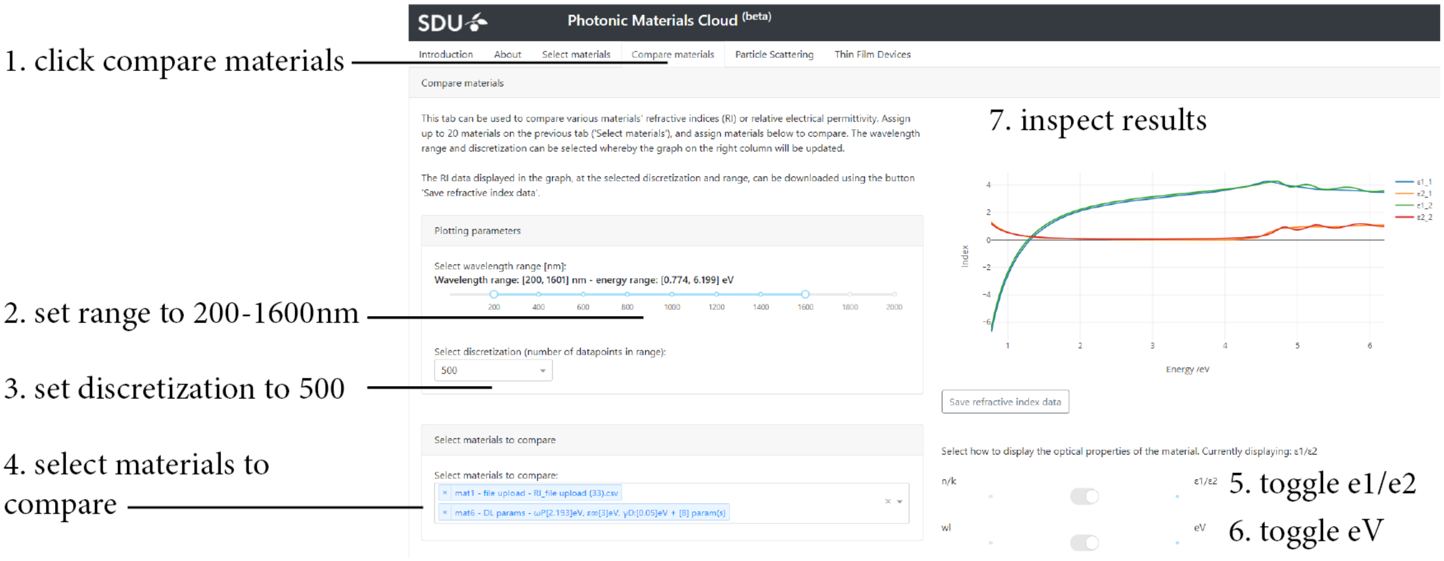

Figure 14: Compare material models of AZO6.

6.2 Mie scattering results, ZrN

This example demonstrates how to assign materials and use the particle scattering tab to to compute Mie scattering results.

6.2.1 Assign materials

First, we need to assign the materials used to demonstrate the Mie calculations results. This example simulates a system of spherical ZrN nanoparticles dispersed in the air. First, the materials should be assigned - unfortunately, the ZrN material is not part of the 'materials library' option on the select materials tab. If the user has access to experimental material nk-data, these can be imported to the dashboard using the 'upload files' option on the select materials tab. However, the user may not have access to experimental data, in which case the 'Drude-Lorentz' option is helpful - in this example, we will demonstrate how to use this option to specify the ZrN material. Also, a medium in which the particles are dispersed must be assigned - this material is air with $n = 1$, $k = 0$. Assign the materials using the photonics materials cloud in the following way.

Table 2: The optical parameters of Drude-Lorentz model for ZrN obtained from DFT-GGA calculations [3]

Resonance j	ω_j [eV]	γ_j [eV]	f_j
1	0	0.62	1
2	0.18	0.24	0.03
3	4.06	0.35	0.02
4	4.75	0.76	0.17
5	5.29	1.26	0.48
6	5.72	0.45	0.09
7	6.62	2.27	1.34
8	7.25	0.40	0.12
9	8.01	1.05	0.55

1. navigate to the select materials tab by clicking 'Select Materials'
2. create and store air medium as material 1 (figure 15):
   - 2.1. click the 'Material with constant refractive index (nk) values' header to unfold the card
   - 2.2. enter $n = 1$ and $k = 0$ for the values for the real- and imaginary part of the refractive index (n- and k value).
   - 2.3. click 'preview material'
   - 2.4. click the drop-down to select, from 1 to 20, which material number the previewed material should be stored. Select 'material 1'
   - 2.5. click 'Assign to material'
3. create and store ZrN material as material 2 (figure 16):
   - 3.1. click the 'Material from Drude-Lorentz parameters' header to unfold the card
   - 3.2. specify the Drude parameters for ZrN - enter $\omega_P = 7.456\,\text{eV}$ as the Drude plasma frequency, $\epsilon_\infty = 1\,\text{F/m}$ as the electric permittivity at the high-frequency limit and $\gamma_D = 0.62\,\text{eV}$ as the Drude damping factor
   - 3.3. choose to specify the Lorentzian peak magnitudes as Lorentzian peak coefficients, f_j
   - 3.4. specify set 1 of Lorentz parameters according to table 2:
     - 3.4.1. fill set (1) of parameters with entries from row no. 2 in table 2:
     - 3.4.2. enter $\omega_1 = 0.18\,\text{eV}$ as the first Lorentzian resonance frequency
     - 3.4.3. enter $\gamma_1 = 0.24\,\text{eV}$ as the first Lorentzian damping constant
     - 3.4.4. enter $f_1 = 0.03$ as the first Lorentzian peak coefficient
   - 3.5. repeat steps 3.4 to fill sets 2-8 of Lorentzian parameters using rows 3-9 in table 2
   - 3.6. click 'preview material'
   - 3.7. click the drop-down to select, from 1 to 20, which material number the previewed material should be stored. Select 'material 2'
   - 3.8. click 'Assign to material'

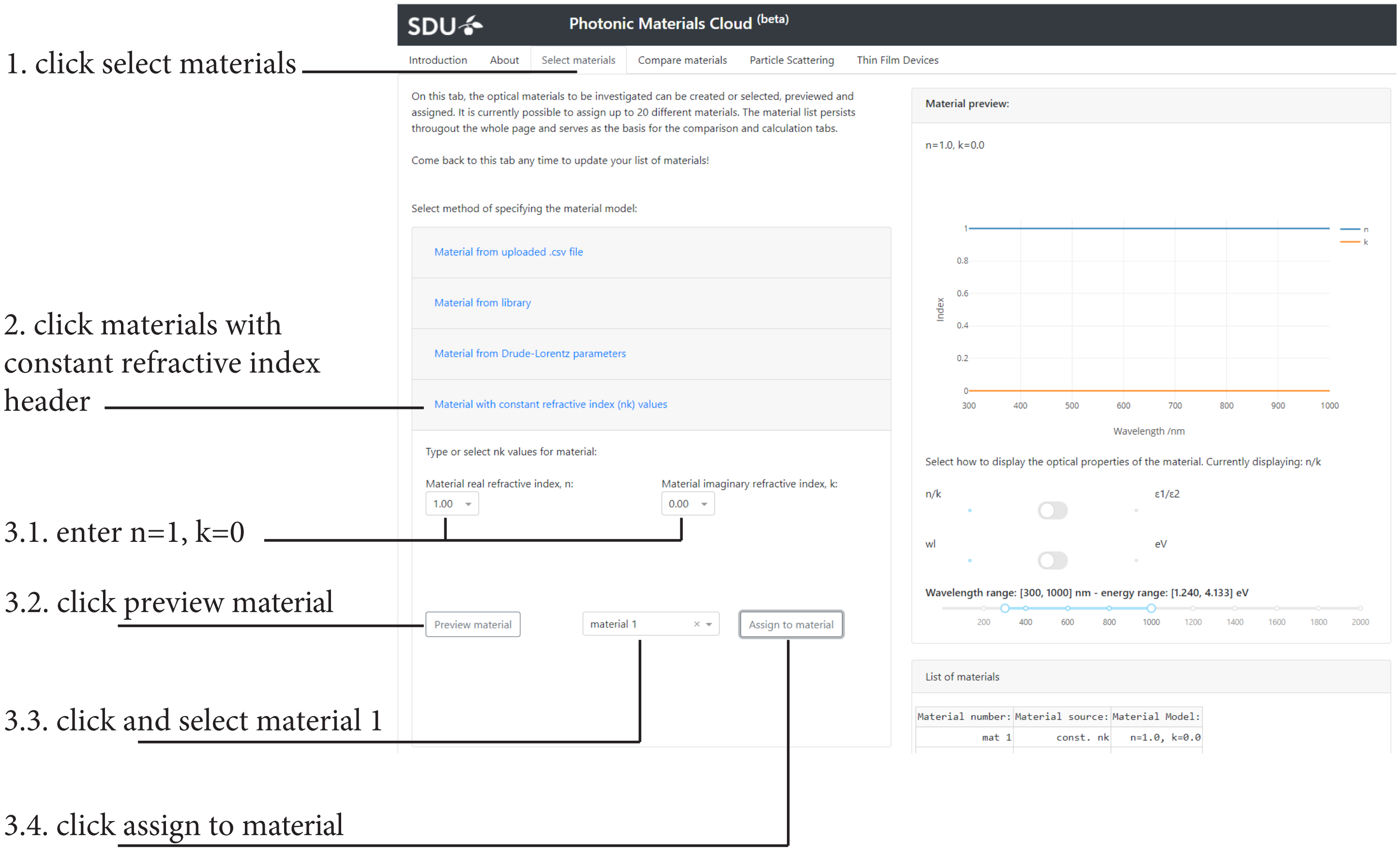

Figure 15: Assign air to the first material of the system.

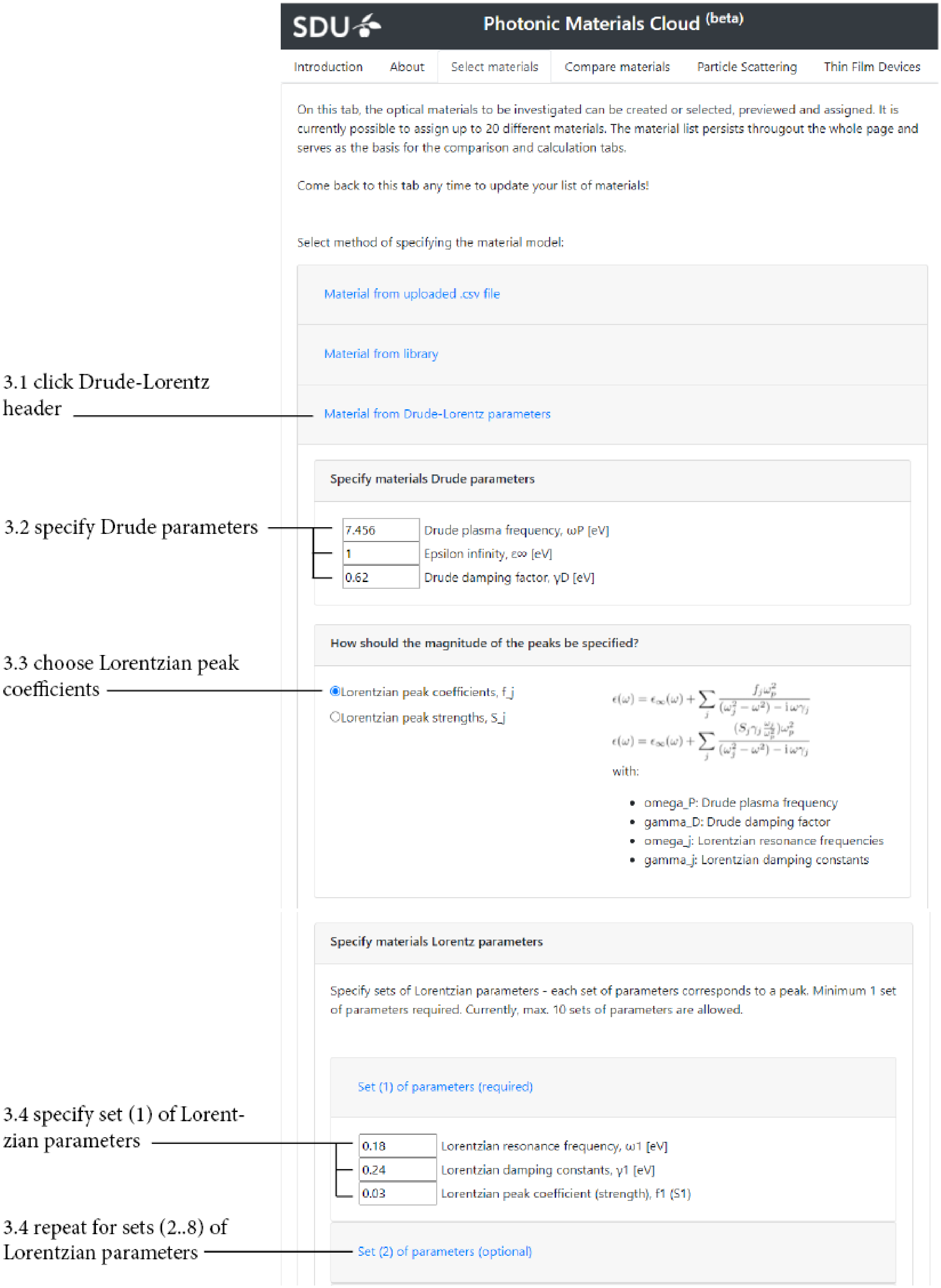

Figure 16: Assign ZrN using Drude-Lorentz parameters to the second material of the system.

6.2.2 Create Dispersed Particle System

Next, the system of dispersed ZrN nanoparticles in air needs to be set up on the dashboard. This is done in the following way (figure 17):

1. navigate to the particle scattering tab by clicking 'Particle Scattering'
2. use the particle core diameter slider to specify the diameter of the spherical nanoparticle to be 65 nm
3. use the shell thickness slider to specify the thickness of the nanoparticle coating to be 0 nm (since there is no coating on the particles in this system)
4. assign materials to the core-, shell- and surrounding medium regions
   - 4.1. click the 'select material of particle core' drop-down and select the ZrN material (mat2 - DL parameters)
   - 4.2. click the 'select material of particle shell' drop-down and select air (mat1 - const. nk). Even though there is no shell, a material must be assigned to the region - in this case, the selection still represents the modelled system since the selected shell material (air) is the same as the medium
   - 4.3. click the 'select material of particle surrounding medium' drop-down and select air (mat1 - const. nk).

6.2.3 Compute Mie-Scattering Results

Finally, the Mie-scattering results are calculated automatically on the dashboard. The resulting scattering data is displayed on the right side of the particle scattering tab, and the wavelength range, discretization and x-axis units can be adjusted by the user. Select the wavelength range γ = 300-1000 eV, discretization of 500 and toggle wavelength for the x-axis unit to generate the plot as displayed in figure 18.

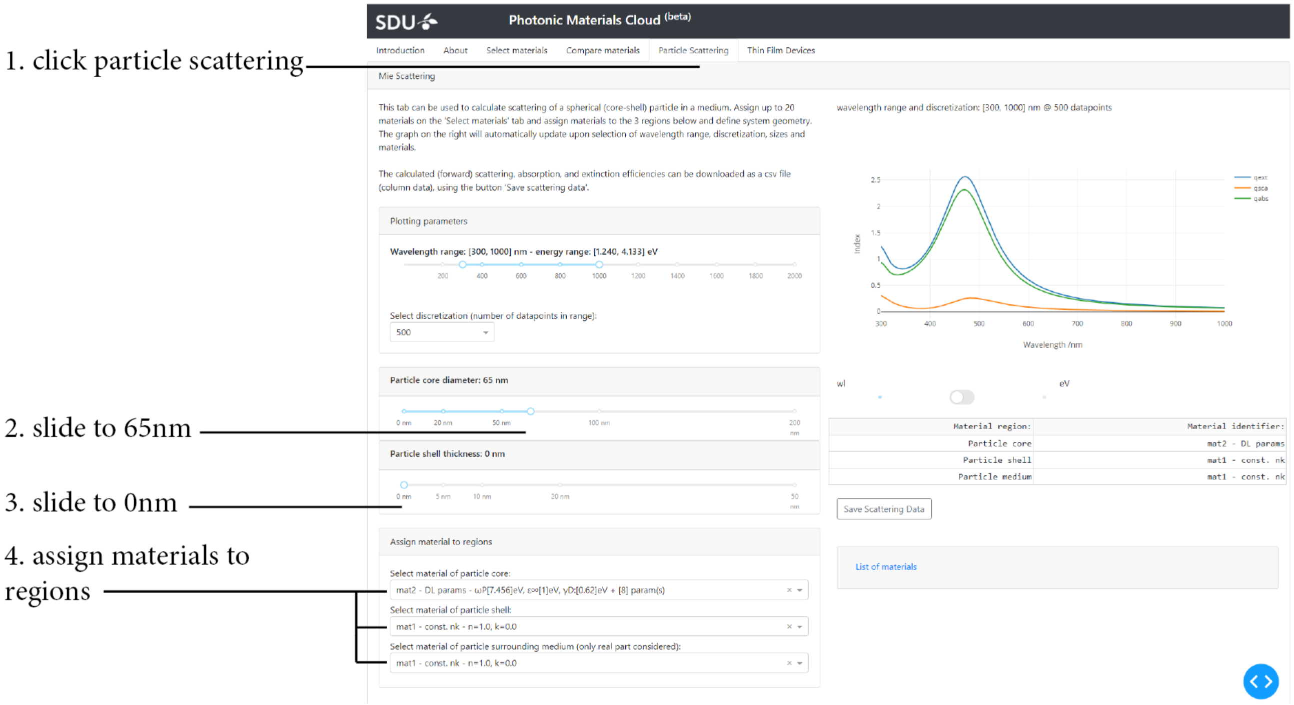

Figure 17: ZrN nanoparticles dispersed in water system setup.

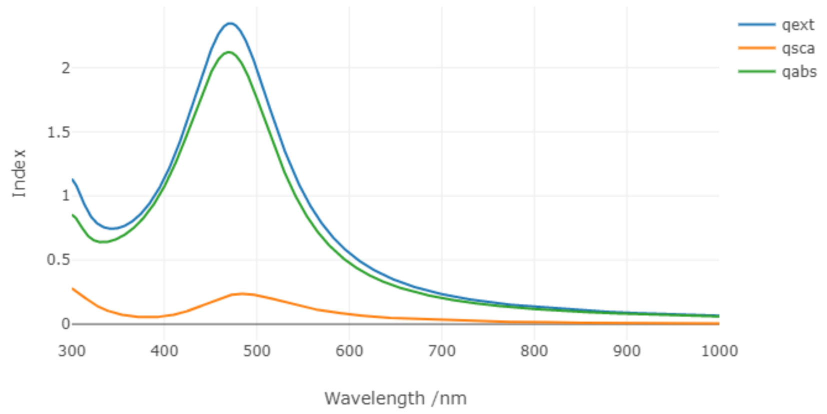

Figure 18: ZrN nanoparticles dispersed in water system - Mie-scattering results.

6.3 Thin Film Results, ZrN

This example demonstrates how to assign materials and use the thin film tab to compute results. The example system is a 50 nm ZrN thin film interfaced with silicon dioxide (SiO_2; $n = 1.52$) and air ($n = 1$). A polychromatic plane wave is an incident through the SiO_2, polarized within the plane of incidence (TM). Then the reflection, transmission and absorption are calculated over a wavelength range of $\lambda = 300$-$800\,\mathrm{eV}$ with the 40-50 degrees incidence angle sweep.

6.3.1 Assign Materials

First, we need to assign the materials used to demonstrate the thin film results. This example simulates a system of ZrN thin-film interfaced with silicon dioxide and air. First, the materials should be assigned - unfortunately, the ZrN material is not part of the 'materials library' option on the select materials tab. If the user has access to experimental material nk-data, these can be imported to the dashboard using the 'upload files' option on the select materials tab. However, the user may not have access to experimental data in which case the 'Drude-Lorentz' option is useful - in this example, we will demonstrate how to use this option to specify the ZrN material. Also, the air-, silicon dioxide media must be assigned. Assign the materials using the photonics materials cloud in the following way.

1. navigate to the select materials tab by clicking 'Select Materials'
2. create and store air medium as material 1 (figure 15):
   - 2.1. click the 'Material with constant refractive index (nk) values' header to unfold the card
   - 2.2. enter $n = 1$ and $k = 0$ for the values for the real- and imaginary part of the refractive index (n- and k value).
   - 2.3. click 'preview material'
   - 2.4. click the drop-down to select, from 1 to 20, which material number the previewed material should be stored. Select 'material 1'
   - 2.5. click 'Assign to material'
3. create and store ZrN material as material 2 (figure 19):
   - 3.1. click the 'Material from Drude-Lorentz parameters' header to unfold the card
   - 3.2. specify the Drude parameters for ZrN - enter $\omega_P = 7.456\,\mathrm{eV}$ as the Drude plasma frequency, $\epsilon_\infty = 1$ [F/m] as the electric permittivity at the high-frequency limit and $\gamma_D = 0.62\,\mathrm{eV}$ as the Drude damping factor
   - 3.3. choose to specify the Lorentzian peak magnitudes as Lorentzian peak coefficients, f_j
   - 3.4. specify set 1 of Lorentz parameters according to table 2:
     - 3.4.1. fill set (1) of parameters with entries from row no. 2 in table 2:
     - 3.4.2. enter $\omega_1 = 0.18\,\mathrm{eV}]$ as the first Lorentzian resonance frequency
     - 3.4.3. enter $\gamma_1 = 0.24\,\mathrm{eV}]$ as the first Lorentzian damping constant
     - 3.4.4. enter $f_1 = 0.03$ as the first Lorentzian peak coefficient

3.5. repeat steps 3.4 to fill sets 2-8 of Lorentzian parameters using rows 3-9 in table 2

3.6. click 'preview material'

3.7. click the drop-down to select, from 1 to 20, which material number the previewed material should be stored. Select 'material 2'

3.8. click 'Assign to material'

4. create and store silicon dioxide medium as material 3 (figure 20):

   4.1. click the 'Material with constant refractive index (nk) values' header to unfold the card

   4.2. enter $n = 1.52$ and $k = 0$ for the values for the real- and imaginary part of the refractive index (n- and k value).

   4.3. click 'preview material'

   4.4. click the drop-down to select, from 1 to 20, which material number the previewed material should be stored. Select 'material 3'

   4.5. click 'Assign to material'

6.3.2 Create Thin Film Layer Sequence

Next, the sequence of layers should be created on the thin film tab. Assign a substrate layer with a thickness of 2500 nm, the ZrN layer and the air medium of impinging wave with 2500 nm thickness using the method described in section 5.3. Generate the layer sequence in the following way (figure 21).

1. navigate to the select materials tab by clicking 'Thin Film Devices'

2. click card header 'add single layer'

3. create and store the air layer as layer 1

   3.1. click the 'select material of TMM layer' drop-down, and select the air material (mat1 - const. nk - $n = 1.0$, $k = 0.0$) by clicking it

   3.2. enter 2500 in the layer thickness box

   3.3. click 'assign as next layer'

4. create and store the ZrN layer as layer 2

   4.1. click the 'select material of TMM layer' drop-down, and select the ZrN material (mat2 - DL params...) by clicking it

   4.2. enter 50 in the layer thickness box

   4.3. click 'assign as next layer'

5. create and store the substrate layer as layer 3

   5.1. click the 'select material of TMM layer' drop-down, and select the substrate refractive index material (mat3 - const. nk - $n = 1.52$, $k = 0.0$) by clicking it

   5.2. enter 2500 in the layer thickness box

   5.3. click 'assign as next layer'

6.3.3 Compute Optical Properties

Finally, the thin film results can be computed calculated in the wavelength- and incidence angle range in which the characteristic resonance is found. The result is computed in the following way (figure 22).

1. click card header 'wavelength sweep, sweep incidence angle' to reveal the card

2. use the wavelength range slider to specify the region of interactive graph to be, Wavelength range: [300 nm, 800 nm] - energy range: [1.550 eV, 4.119 eV]

3. use the toggle button to select TM (transverse magnetic field polarization of the impinging wave)

4. specify the discretization of the interactive graph to be 500 datapoints

5. use the incidence angle range slider to specify the region of interactive graph to be, Incidence angle range: [40 deg, 50 deg]
6. specify the discretization of the interactive graph to be 100 datapoints
7. use the toggle button to select the contour plot option
8. click calculate
9. inspect the results
10. click save optical data

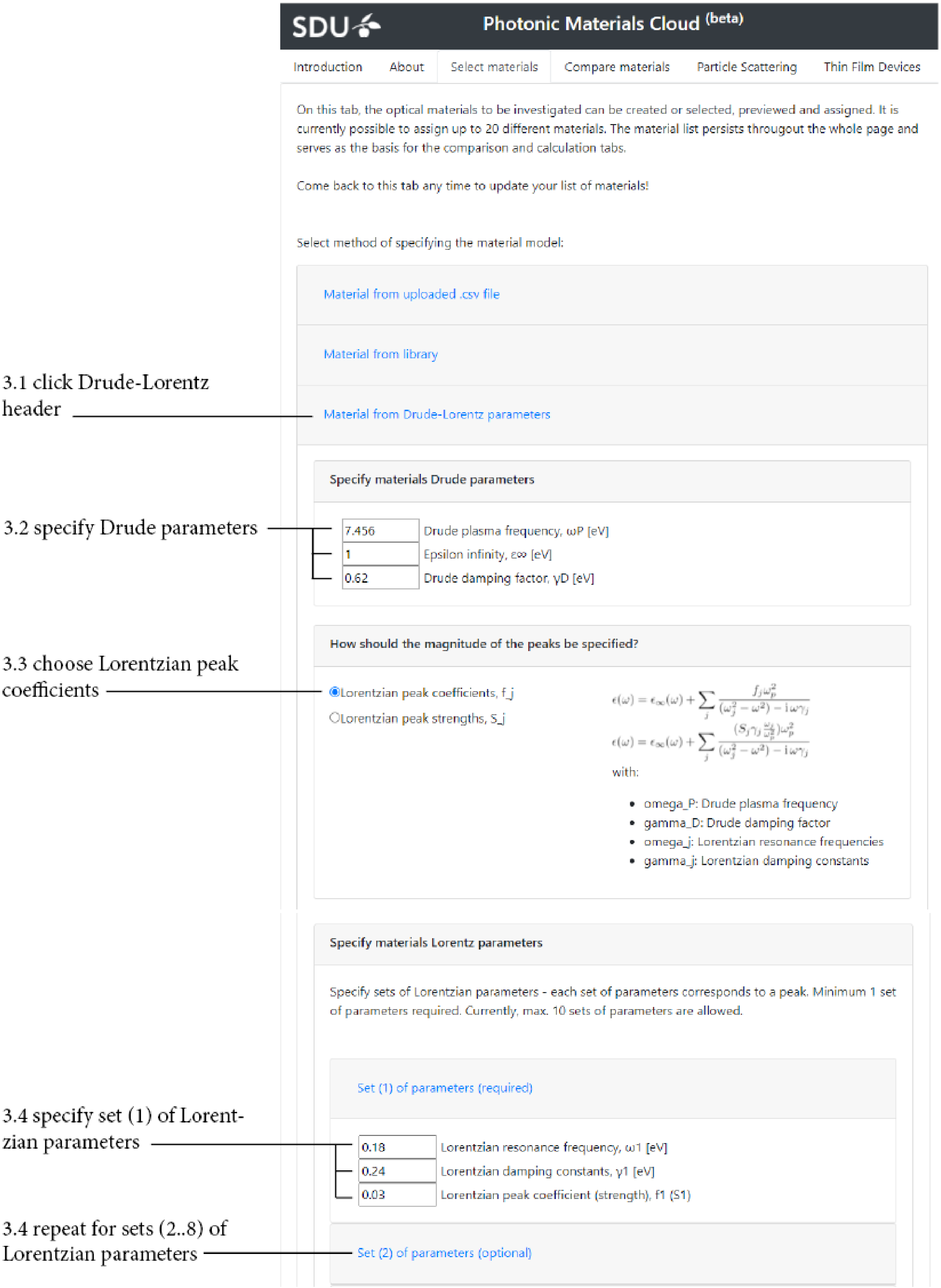

Figure 19: Assign ZrN using Drude-Lorentz parameters to the second material of the system.

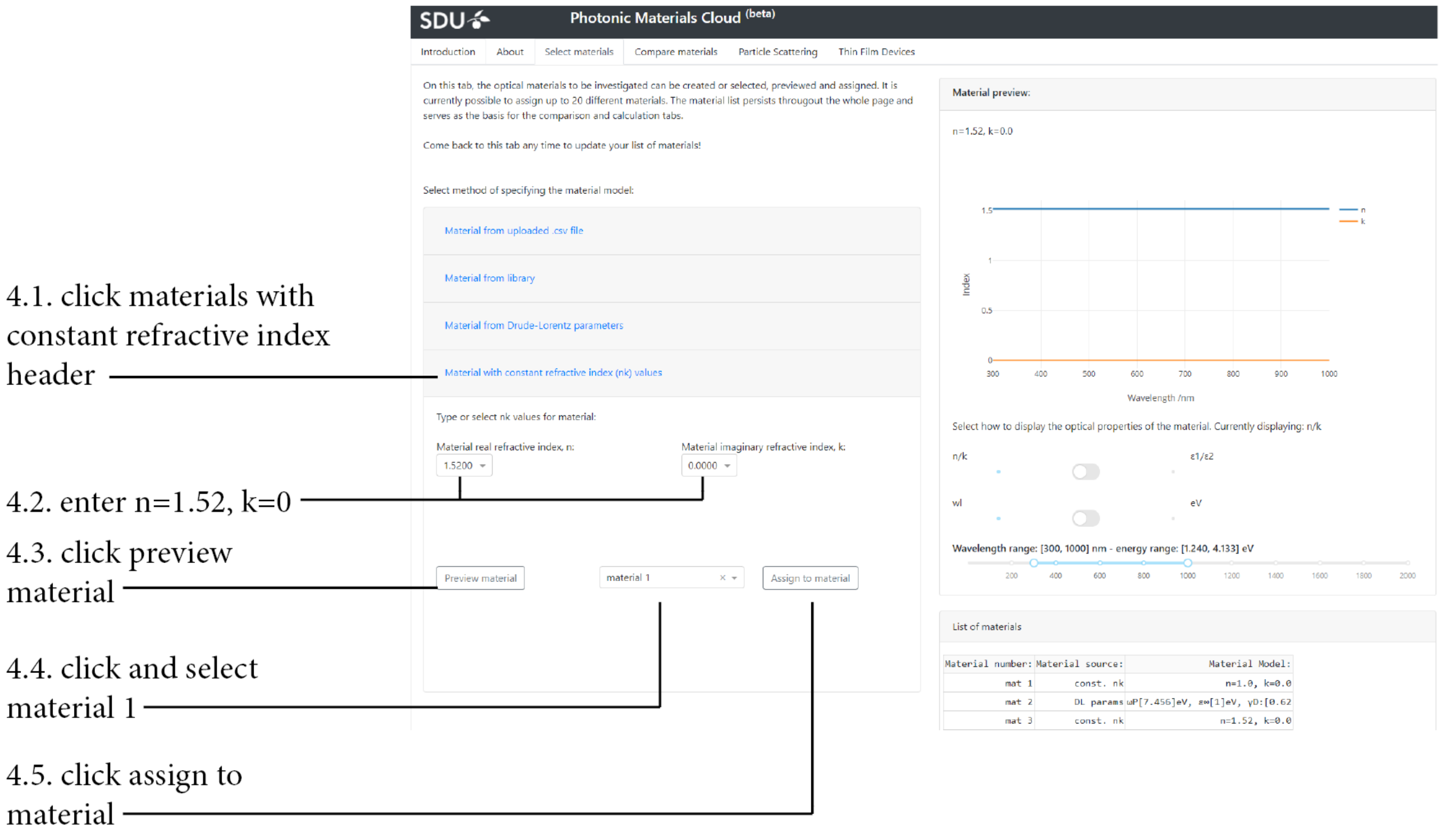

Figure 20: Assign silicon dioxide to the third material of the thin film system.

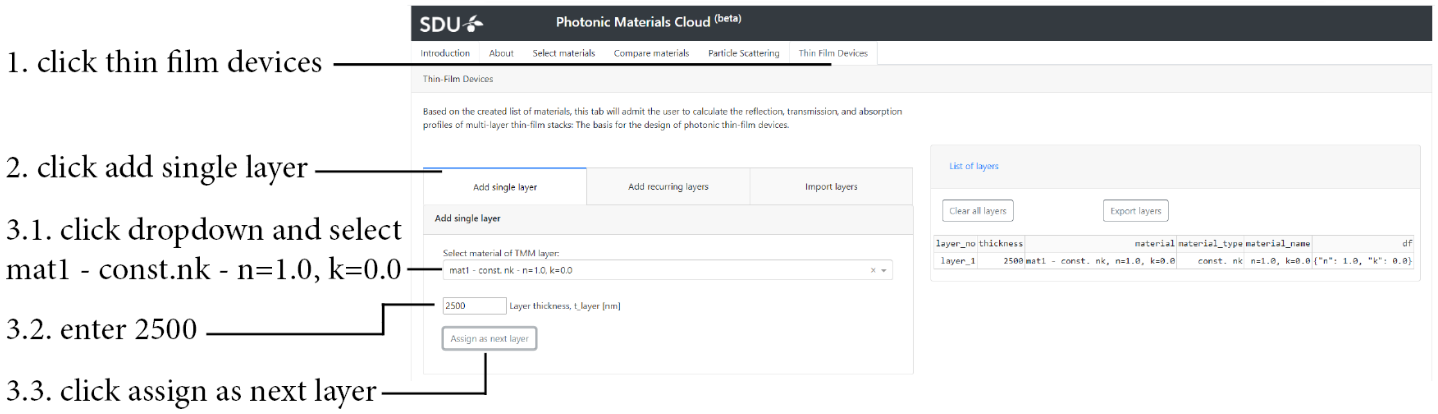

Figure 21: Define thin film layers.

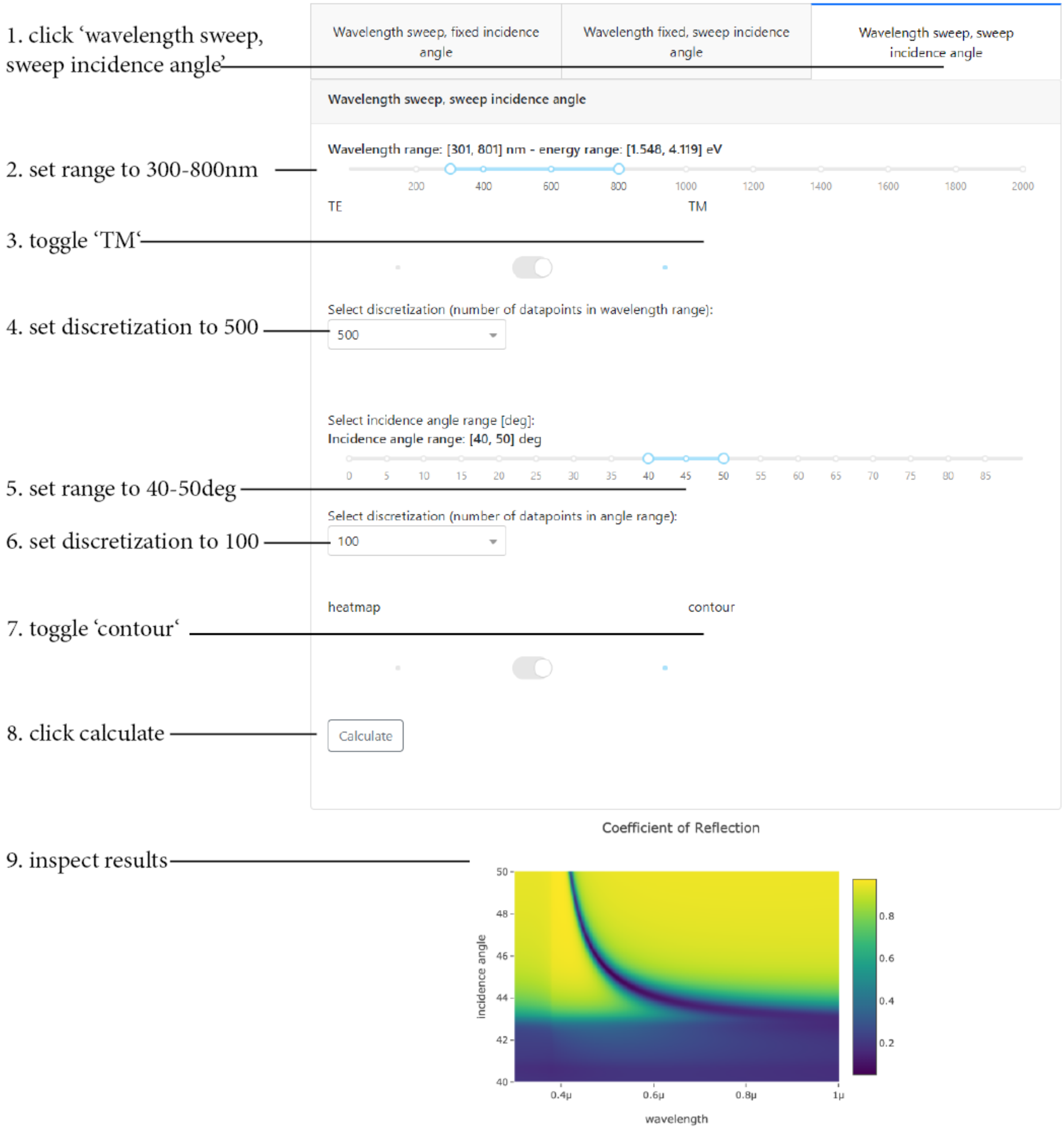

Figure 22: Generate 2D results of the thin film system.

6.4 The Bragg Reflector

This example demonstrates how to assign materials and use the thin film tab to generate recurring layers as well as compute results.

6.4.1 Assign materials

First, we need to assign the materials used to demonstrate the Bragg reflector. The Bragg reflector works by having alternating layers of higher- and lower refractive index media placed on a substrate. Also, a medium for the impinging wave must be assigned - air is used with $n = 1$. Assign the materials using the photonics materials could in the following way.

1. navigate to the select materials tab by clicking 'Select Materials'
2. click the 'Material with constant refractive index (nk) values' header to unfold the card
3. create and store air medium as material 1 (figure 15):
   3.1. enter $n = 1$ and $k = 0$ for the values for the real- and imaginary part of the refractive index (n- and k value).
   3.2. click 'preview material'
   3.3. click the drop-down to select, from 1 to 20, which material number the previewed material should be stored. Select 'material 1'
   3.4. click 'Assign to material'
4. create and store higher refractive index medium as material 2:
   4.1. enter $n = 3.49$ and $k = 0$ for the values for the real- and imaginary part of the refractive index (n- and k value).
   4.2. click 'preview material'
   4.3. click the drop-down to select, from 1 to 20, which material number the previewed material should be stored. Select 'material 2'
   4.4. click 'Assign to material'
5. create and store lower refractive index medium as material 3:
   5.1. enter $n = 2.95$ and $k = 0$ for the values for the real- and imaginary part of the refractive index (n- and k value).
   5.2. click 'preview material'
   5.3. click the drop-down to select, from 1 to 20, which material number the previewed material should be stored. Select 'material 3'
   5.4. click 'Assign to material'
6. create and store substrate medium as material 4:
   6.1. enter $n = 1.52$ and $k = 0$ for the values for the real- and imaginary part of the refractive index (n- and k value).
   6.2. click 'preview material'
   6.3. click the drop-down to select, from 1 to 20, which material number the previewed material should be stored. Select 'material 4'
   6.4. click 'Assign to material'

6.4.2 Create Bragg reflector layer sequence

Next, the sequence of layers in the Bragg reflector should be created on the thin film tab. Assign a substrate layer with a thickness of 2500 nm, the 25 recurring layers of higher refractive index material with 71.6 nm thickness and lower refractive index material with 84.7 nm, then finally the air medium of impinging wave with 2500 nm thickness. Since the recurring layers assignment tab is not yet implemented, the sequence is generated using the method described in section 5.3. Generate the Bragg reflector sequence in the following way.

1. navigate to the thin-film tab by clicking 'Thin Film Devices'
2. click card header 'add single layer'
3. create and store the air layer as layer 1 (figure 23)
   3.1. click the 'select material of TMM layer' drop-down and select the air material (mat1 - const. nk - $n = 1.0$, $k = 0.0$) by clicking it

3.2. enter 2500 in the layer thickness box

3.3. click 'assign as next layer'

4. create and store a higher refractive index layer as layer 2

4.1. click the 'select material of TMM layer' drop-down and select the higher refractive index material (mat2 - const. nk - $n = 3.49$, $k = 0.0$) by clicking it

4.2. enter 71.6 in the layer thickness box

4.3. click 'assign as next layer'

5. create and store a lower refractive index layer as layer 3

5.1. click the 'select material of TMM layer' drop-down and select the lower refractive index material (mat3 - const. nk - $n = 2.95$, $k = 0.0$) by clicking it

5.2. enter 84.7 in the layer thickness box

5.3. click 'assign as next layer'

6. create and store the substrate layer as layer 4

6.1. click the 'select material of TMM layer' drop-down and select the substrate refractive index material (mat4 - const. nk - $n = 1.52$, $k = 0.0$) by clicking it

6.2. enter 2500 in the layer thickness box

6.3. click 'assign as next layer'

7. click the 'List of Layers' header

8. click 'Export Layers'

9. open the downloaded .csv file using a text editor

10. copy layers 2 and 3 and paste them 20 times, between the substrate and air medium, so that the number of recurring layers is 20 and the total number of layers is 52 (figure 24)

11. save the file with the copied layers to a new .csv file. It is not necessary to rename the index- or layer number as this will be done automatically in the dashboard once the file is imported

12. go back to the dashboard and click card header 'import layers' to reveal the card

13. click the 'Drag and Drop or Select Files' to open the dialog window

14. navigate to the previously saved .csv file containing the Bragg sequence of layers and select it by clicking it

15. click the 'import layers' button to load the sequence of layers into the dashboard (figure 25)

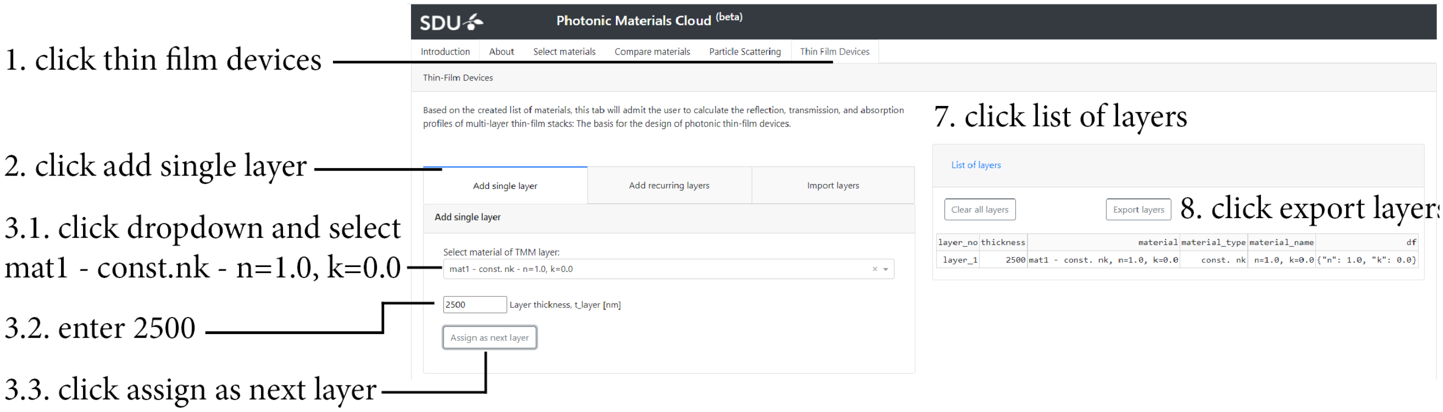

Figure 23: Define unique layers of the Bragg reflector.

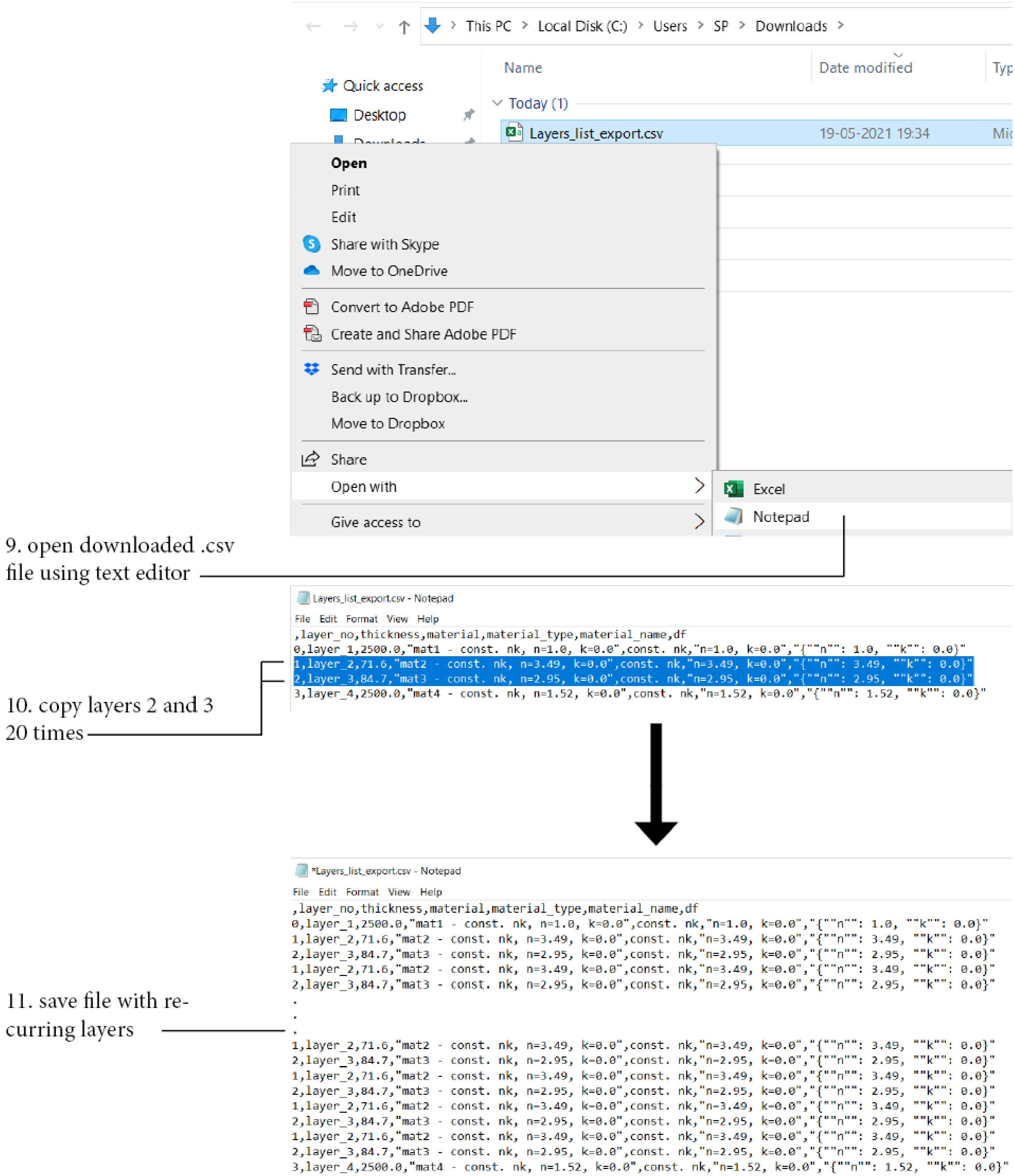

Figure 24: Copy and save unique layers of the Bragg reflector to new .csv file.

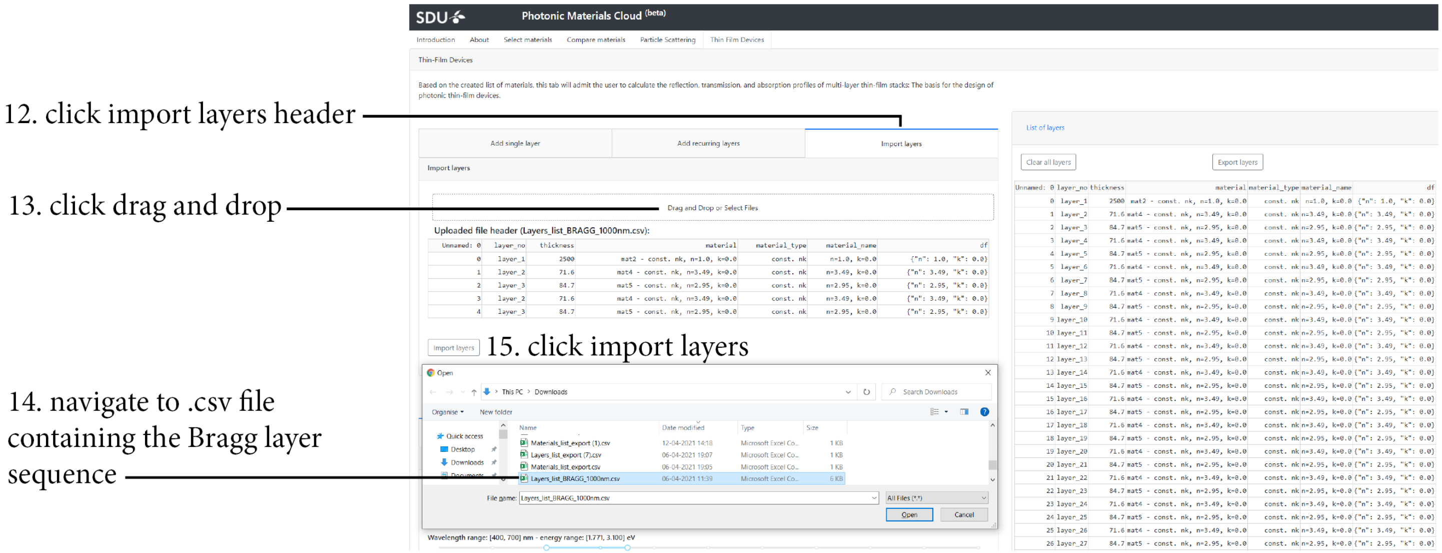

Figure 25: Import the .csv file containing the recurring layers of the Bragg reflector.

6.4.3 Compute optical properties

Finally, the Bragg reflectors optical properties can be computed using the generated thin-film device.
The wavelength range with total reflectance at a specific wave incidence angle of the Bragg reflector can be demonstrated by computing a 'wavelength sweep, fixed incidence angle' result. The wavelength region of total reflection for the Bragg reflector in this example is $\lambda \sim$ 950-1050 nm - this result is computed in the following way (figure 26).

1. click card header 'wavelength sweep, fixed incidence angle' to reveal the card
2. use the toggle button to select wl (wavelength) or eV (electron-volt) for the unit of the interactive graph plotting the optical data
3. use the wavelength range slider to specify the region of interactive graph to be, Wavelength range: [900 nm, 1200 nm] - energy range: [1.032 , 1.379 eV]
4. use the toggle button to select TE (transverse electric field polarization of the impinging wave)
5. specify the incidence angle of the impinging wave to be 0 deg
6. specify the discretization of the interactive graph to be 500 datapoints
7. click calculate
8. inspect the results
9. click save optical data

Figure 26 (8) illustrates how the example Bragg reflector has total reflectance in the wavelength region $\lambda \sim$ 950-1050 nm for a TE wave with incidence angle of 0 deg.
The wavelength range with total reflectance is dependant on wave incidence the angle of the Bragg reflector; why the wavelength region shifts with changing incidence angle - this interdependence can be demonstrated by computing a 'wavelength sweep, sweep incidence angle' result. In this Bragg reflector example, the region investigated is the wavelengths $\lambda \sim$ 700-1200 nm and incidence angles $\theta \sim$ 0-70 degree - this result is computed in the following way (figure 27).

1. click card header 'wavelength sweep, sweep incidence angle' to reveal the card
2. use the wavelength range slider to specify the region of interactive graph to be, Wavelength range: [700 nm, 1200 nm] - energy range: [1.031 eV, 1.774 eV]
3. use the toggle button to select TE (transverse electric field polarization of the impinging wave)
4. specify the discretization of the interactive graph to be 500 datapoints
5. use the incidence angle range slider to specify the region of interactive graph to be 'Incidence angle range: [0 deg, 70 deg]
6. specify the discretization of the interactive graph to be 100 datapoints
7. use the toggle button to select the heatmap plot option
8. click calculate
9. inspect the results
10. click save optical data

Figure 27 (9) illustrates how the example Bragg reflector has total reflectance in the wavelength region $\lambda \sim$ 950-1050nm for a TE wave with an incidence angle of 0 deg but the region of total reflection blue-shifts with increasing incidence angle.

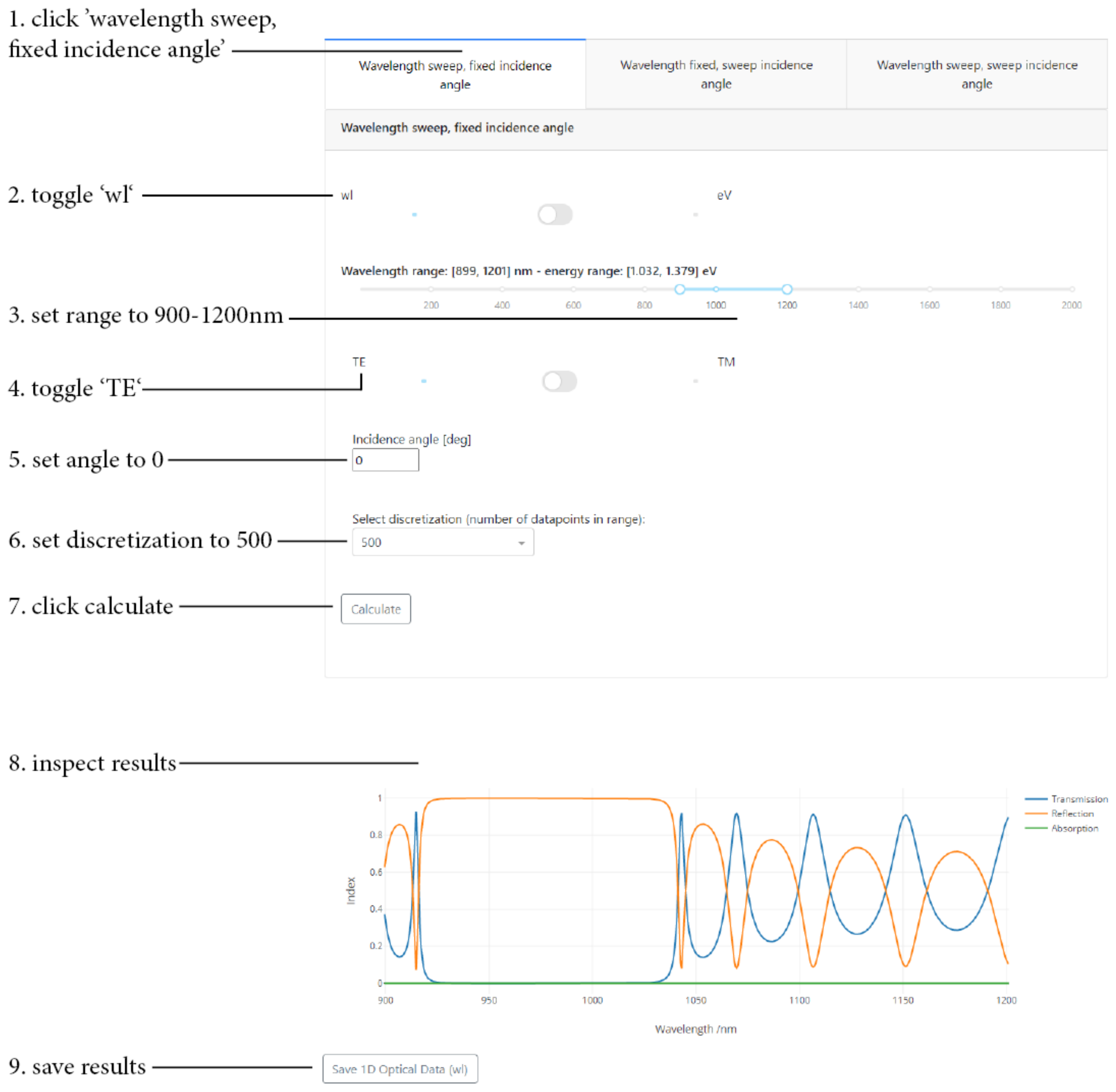

Figure 26: Generate 1D results of the Bragg reflector.

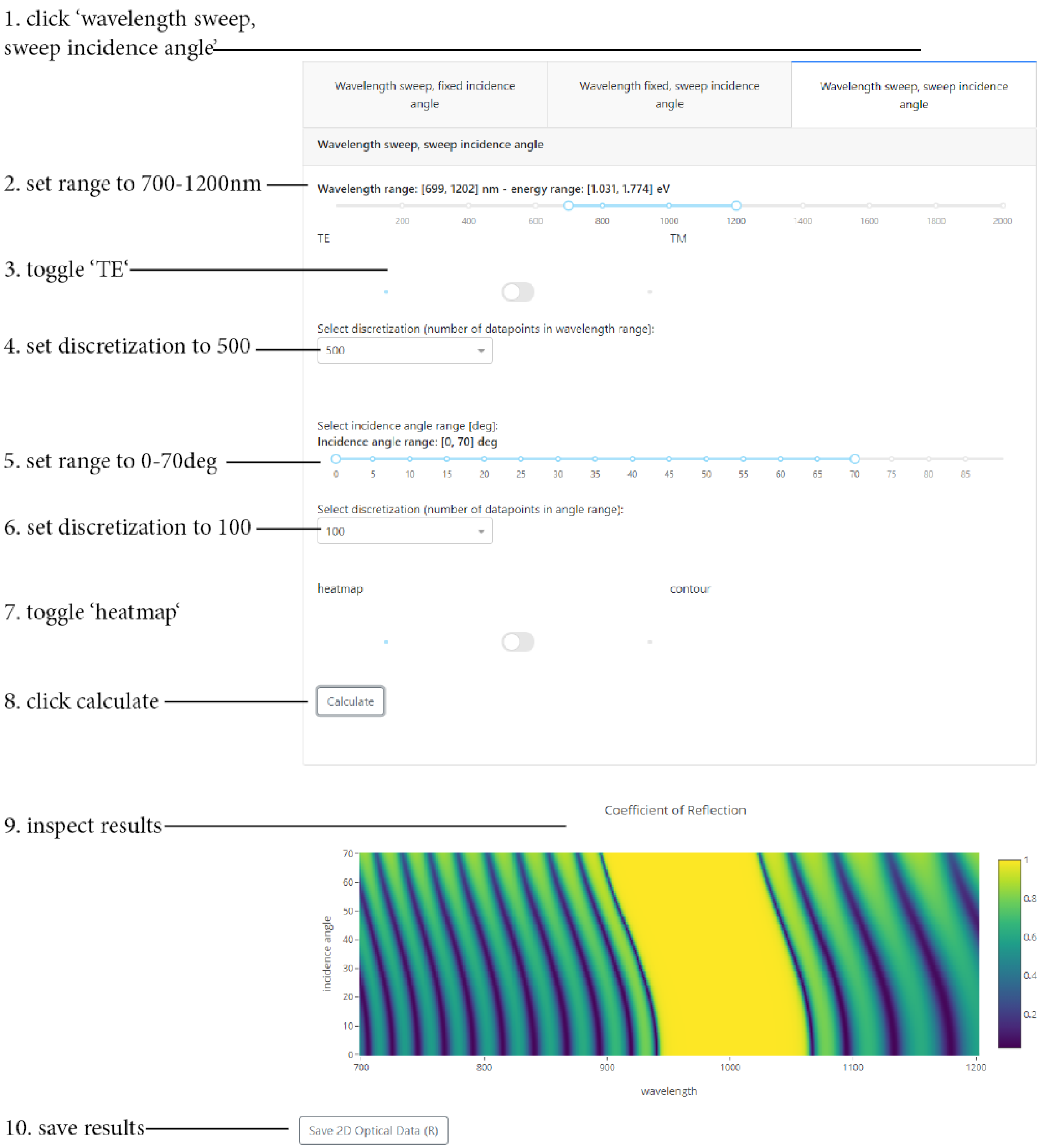

Figure 27: Generate 2D results of the Bragg reflector.

References

[1] The computational materials group at sdu. `https://sdu.dk/compmat`. Accessed on 2022-07-04.

[2] The photonic materials cloud. `http://photonicmaterials.eu`. Accessed on 2022-07-04.

[3] Alireza Shabani, Matiyas Tsegay Korsa, Søren Petersen, Mehdi Khazaei Nezhad, Yogendra Kumar Mishra, and Jost Adam. Zirconium nitride: Optical properties of an emerging intermetallic for plasmonic applications. *Advanced Photonics Research*, page 202100178, 2021.
